# Characterization of the SARS-CoV-2 Genome 3′-Untranslated Region Interactions with Host MicroRNAs

**DOI:** 10.1021/acsomega.4c01050

**Published:** 2024-08-16

**Authors:** Caleb J. Frye, Caylee L. Cunningham, Mihaela Rita Mihailescu

**Affiliations:** Department of Chemistry and Biochemistry, Duquesne University, Pittsburgh, Pennsylvania 15282, United States

## Abstract

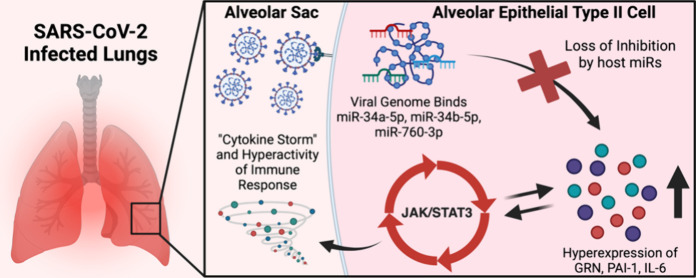

The 2019 pandemic, caused by severe acute respiratory syndrome coronavirus 2 (SARS-CoV-2), has marked the spread of a novel human coronavirus. SARS-CoV-2 has exhibited increased disease severity and immune evasion across its variants, and the molecular mechanisms behind these phenomena remain largely unknown. Conserved elements of the viral genome, such as secondary structures within the 3′-untranslated region (UTR), could prove crucial in furthering our understanding of the host–virus interface. Analysis of the SARS-CoV-2 viral genome 3′-UTR revealed the potential for host microRNA (miR) binding sites, allowing for sequence-specific interactions. In this study, we demonstrate that the SARS-CoV-2 genome 3′-UTR binds the host cellular miRs miR-34a-5p, miR-34b-5p, and miR-760-3p *in vitro*. Native gel electrophoresis and steady-state fluorescence spectroscopy were utilized to biophysically characterize the binding of these miRs to their predicted sites within the SARS-CoV-2 genome 3′-UTR. Additionally, we investigated 2′-fluoro-d-arabinonucleic acid (FANA) analogs as competitive binding inhibitors for these interactions. These miRs modulate the translation of granulin (GRN), interleukin-6 (IL-6), and the IL-6 receptor (IL-6R), all of which are key modulators and activators of JAK/STAT3 signaling and are implicated in regulation of the immune response. Thus, we propose that hijacking of these miRs by SARS-CoV-2 could identify a mechanism of host immune modulation by the virus. The mechanisms detailed in this study have the potential to drive the development of antiviral treatments for SARS-CoV-2, through direct targeting of the virus–host interface.

## Introduction

Severe acute respiratory syndrome coronavirus 2 (SARS-CoV-2), the virus responsible for the coronavirus disease 2019 (COVID-19) pandemic, has caused more than 7.1 million deaths as of August 2024.^1^ Development of the mRNA vaccines to combat SARS-CoV-2 has mitigated these numbers; however, the virus still poses a threat to those who are immunocompromised and/or have underlying health issues.^2^ Additional treatment options do exist, but the molecular mechanisms behind disease severity and the viral life cycle remain largely unknown, highlighting the need for further characterization of the virus. The single-stranded RNA (ssRNA) SARS-CoV-2 genome is 29,903 nucleotides (nt) in length and harbors both 5′- and 3′-UTRs, which have been shown to play a key role in viral replication through the conserved RNA secondary structural elements.^3^ In addition to their own machinery, viruses are known to hijack host elements to promote viral fitness, including microRNAs (miRs).^4^ Interestingly, it has been shown that miRs hijacked by viruses are predominantly involved in inflammatory or immune signaling pathways, suggesting that hijacking of immune miRs could be generally beneficial.^5,6^

Severe acute respiratory syndrome coronavirus (SARS-CoV), the virus responsible for the 2002–2003 SARS outbreak, has been shown to alter expression of miRs that regulate immune cell proliferation and activation, as well as the expression of the angiotensin converting enzyme 2 (ACE2) receptor that is used for entry into the cell.^7−9^ This has been demonstrated in bronchoalveolar stem cells and supports the binding of multiple host miRs to the viral genome, across both coding and noncoding regions.^9^ Since SARS-CoV is an evolutionary predecessor to SARS-CoV-2, we and others predicted that the virus can undergo similar interactions with host miRs.^10,11^ Computational analysis of the SARS-CoV-2 genome revealed hundreds of potential miR-RNA binding interactions, centered around conserved secondary structural elements and on the viral genome 3′-UTR.^12,13^ Similarly to SARS-CoV and other RNA viruses, it was found that SARS-CoV-2 can interact with host miRs involved in the immune response.^12^ One conserved secondary structure, the stem loop II-like motif (s2m), has been shown to be involved in homodimerization; this element was also shown to bind two copies of host miR-1307-3p.^14^ MiR-1307-3p has been predicted to regulate a large variety of cytokines and their receptors, highlighting its significance in the immune response.^14^ The binding of a host miR to the viral genomic RNA could indirectly modulate host cell translation, highlighting the significance of these interactions. Scheel et al. have demonstrated that at least 15 viruses, including the chikungunya virus, bovine viral diarrhea virus, and respiratory syncytial virus, among others, can sequester both argonaute (AGO)-loaded miRs and free miRs for the benefit of the viral life cycle.^15^ Specifically, the bovine viral diarrhea virus was found to require the binding of miR-17 for replication, highlighting that the binding of the miR, which normally acts to inhibit translation or trigger degradation of the target RNA, can act otherwise to be beneficial to the virus.^15^

**Figure 1:**
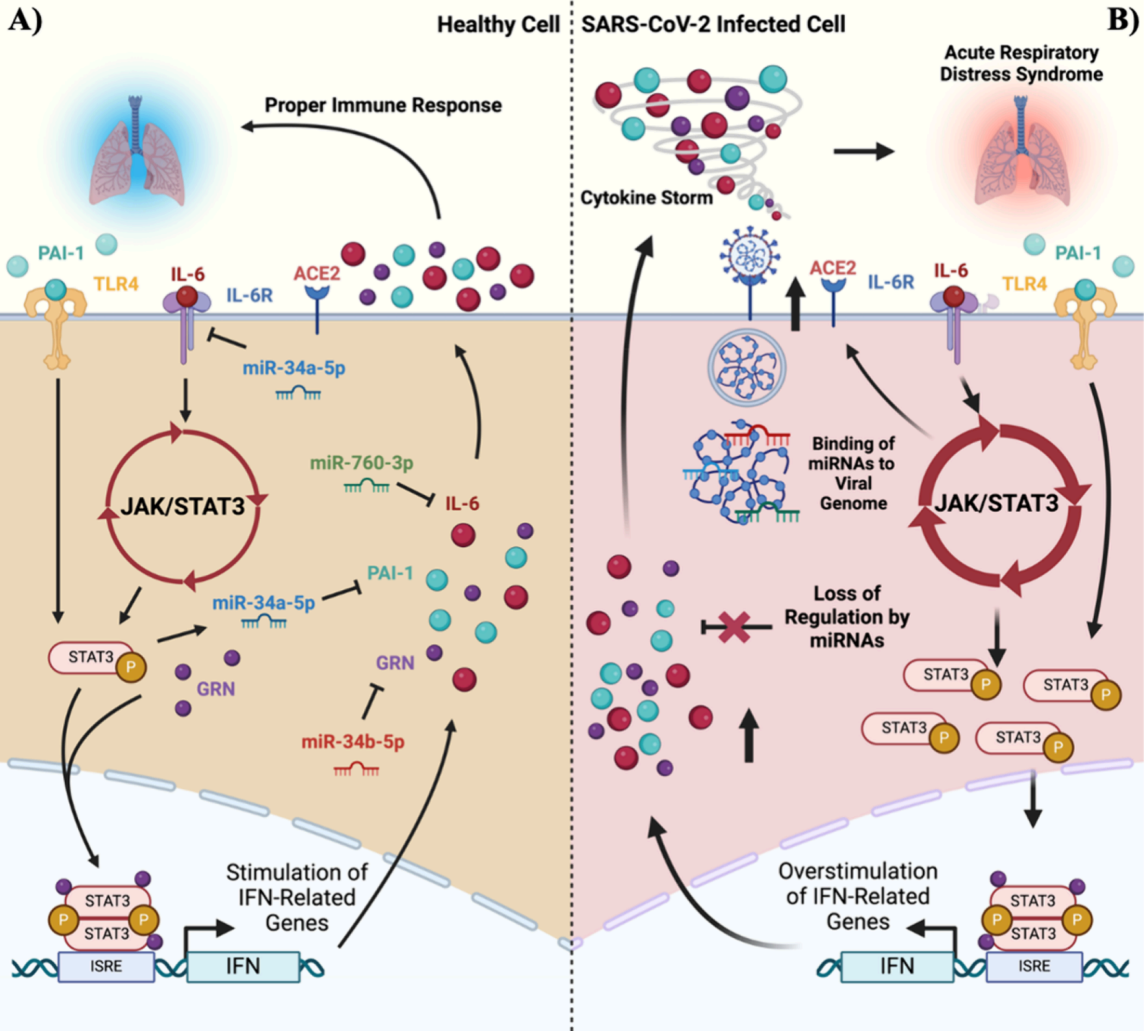
Roles of miR-34a-5p, miR-34b-5p, and miR-760-3p in JAK/STAT3 regulation. (A) In a healthy cell, JAK/STAT3 signaling can be activated by the binding of IL-6 and other interleukins to their respective receptors. Activation of this pathway triggers phosphorylation of STAT3, which then homodimerizes and associates with GRN. This complex binds to the IFN-related genes, stimulating production of PAI-1, GRN, and IL-6, and the ACE2 receptor. These proteins then act in an autocrine or paracrine manner, triggering positive feedback cycles for JAK/STAT3 activation. PAI-1, GRN, and IL-6 are regulated by host miR-34a-5p, miR-34b-5p, and miR-760-3p, respectively, which prevent overstimulation of JAK/STAT3. MiR-34a-5p can also regulate IL-6R for a similar effect in the regulation of JAK/STAT3. (B) Infection by SARS-CoV-2 and the proposed subsequent hijacking of host miR-760-3p, miR-34a-5p, and miR-34b-5p by the viral RNA could remove the regulation of PAI-1, GRN, and IL-6, triggering uncontrolled activation cycles of JAK/STAT3. This overactivation can result in the mass production of cytokines and pro-inflammatory molecules, triggering the “cytokine storm” and ARDS, while also producing an excess of the ACE2 receptor and allowing greater viral entry into the cell. Created with BioRender.com.

Fossat et al. employed cross-linking immunoprecipitation combined with RNA proximity ligation (CLEARCHIP) in SARS-CoV-2 infected VeroE6 and A549hACE2 cells, identifying hundreds of host miRs bound to the viral RNA *in vivo*.^16^ A significant number of miRs identified by Fossat et al. are involved in Toll-like receptor (TLR) activation and cytokine production, as well as in apoptosis inhibition.^16^ In this study, we focused on miR-34a-5p, miR-34b-5p, and miR-760-3p, which bind to SARS-CoV-2 RNA *in vivo* and play key roles in the regulation of various steps in the JAK/STAT pathway, highlighting their significance in immune regulation (Figure 1A).^16^ MiR-34a-5p has been shown to regulate plasminogen activator inhibitor-1 (PAI-1), which activates STAT3 through the Toll-like Receptor 4 (TLR4) signaling cascade.^17^ This miR also directly regulates the translation of the IL-6 receptor.^17−21^ MiR-34b-5p has been shown to regulate the translation of granulin (GRN), which activates cytokine production by assisting STAT3 in its role as a transcription factor for the expression of interferon (IFN)-related genes.^22−26^ Lastly, miR-760-3p has been shown to regulate the translation of both IL-6 and the ACE2 receptor.^27−30^ Each of these miRs are expressed in lung epithelial cells^31−33^ and exhibit varied expression profiles upon SARS-CoV-2 infection, which is expected, given their role in the host immune response.^7,34^ In lung tissue biopsy samples and in the blood serum of SARS-CoV-2 patients, expression levels of miR-34a-5p and miR-760-3p were found to be elevated, especially in severe and moderate cases with a 1.5×–1.6×-fold increase in expression, respectively.^34−36^ Meanwhile, miR-34b-5p was found to be downregulated by nearly 57% upon infection with SARS-CoV-2.^34−36^ Similar trends in the expression of these miRs have been reported, with increased miR-34a-5p and miR-760-3p and decreased miR-34b-5p expression levels identified in the hepatitis B virus and respiratory syncytial virus infected cells, supporting their role in the host immune response.^37−39^

Despite the fluctuation in miR levels upon infection, the host mRNA targets of these miRs are all upregulated in SARS-CoV-2 infection.^25,40,41^ This suggests an uncoupling of regulation of GRN, IL-6, IL-6R, and PAI-1 by their respective miRs upon infection and implies that the SARS-CoV-2 virus may be involved. Overstimulation of the immune response by a miR binding would seem detrimental to the virus; however, by binding to the miRs and removing the inhibitory control of multiple checkpoints in JAK/STAT3, overactivation could be favorable due to increased ACE2 expression.^40,42^ JAK/STAT3 signaling is also known to activate and polarize macrophages, which express ACE2, in which overactivation of the immune response could localize macrophages to the infected cells, ultimately bolstering infectivity in both lung epithelial cells and macrophages.^43,44^

Considering the role of miR-34a-5p, miR-34b-5p, and miR-760-3p in the regulation of the JAK/STAT3 pathway, we hypothesized that the SARS-CoV-2 genome 3′-UTR binds these miRs with high affinity, potentially “sponging” them and leading to deregulated JAK/STAT3 signaling and increased ACE2 expression (Figure 1B). Moreover, we hypothesize that the miR interactions with the SARS-CoV-2 3′-UTR can be inhibited by 2′-fluoro-d-arabinonucleic acid (FANA) antisense oligonucleotides. We speculate that these interactions establish a mechanism of therapeutic intervention between host miRs and SARS-CoV-2 viral RNAs.

## Results and Discussion

**Figure 2:**
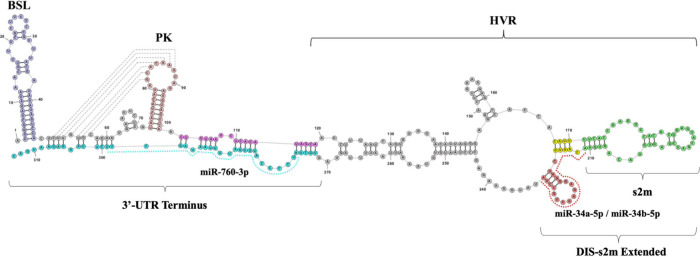
Schematic diagram of the SARS-CoV-2 viral genome 3′-UTR highlighting structural elements. The secondary structure of the 3′-UTR, as predicted by RNAstructure and StructureEditor software packages, was used as a structural basis for miRNA binding site predictions. The bulged stem loop (BSL, purple) and pseudoknot (PK, salmon) are indicated. Host cellular microRNAs and their binding sites are indicated by dashed lines adjacent to the colored binding sites: miR-760-3p, turquoise; miR-34a-5p, miR-34b-5p, red; HVR, hypervariable region.

We and others have analyzed the SARS-CoV-2 3′-UTR (reference genome NCBI: NC_045512.2) for potential binding sites for host miRs and identified that miR-34a-5p and miR-34b-5p share the same predicted binding site downstream of, and into the lower stem of, s2m (nt 29,768–29,790; Figure 2, nt 211–233, red dashed line). For miR-760-3p we identified a predicted binding site at the end of the 3′-UTR (nt 29,833–29,856; Figure 2, nt 276–299, turquoise dashed line).^10,14,45−48^ To test if these miRs bind to their predicted sites, we first used model systems composed of short oligonucleotides that mimic the respective binding site, followed by the analysis of these interactions in the context of the full-length SARS-CoV-2 3′-UTR.

### Host miR-34a-5p and miR-34b-5p Bind Downstream of the SARS-CoV-2 Genome 3′-UTR s2m Element

The predicted binding site for host miR-34a-5p and miR-34b-5p folds into a short hairpin structure (Figure 2, red) immediately downstream of the extended s2m element (Figure 2, green and yellow).^14,45^ Thus, to match the secondary structure of the proposed binding site, we used a construct which contains s2m and its extended lower stem (Figure 2, green and yellow) and the predicted miR binding site (Figure 2, red), which we named here the dimer initiation site-s2m extended (DIS-s2m extended).

Prior to our analysis of the miR binding interactions, we tested the dimerization of the DIS-s2m extended, given that the s2m itself dimerizes in the presence of Mg^2+^ through the formation of a kissing dimer, which converts to an extended duplex structure, affecting its migration in native PAGE experiments.^14^

Thus, we compared the full-length DIS-s2m extended construct (Figure 2, green, yellow, and red), the isolated s2m (Figure 2, green), and s2m with its extended stem, called here DIS-s2m (Figure 2, green, and yellow) for their dimerization properties (Figure S1A). When incubated in the presence of increasing Mg^2+^ concentrations in a tris boric acid with 5 mM MgCl_2_ (TBM) gel, the isolated s2m forms a mixture of monomer, kissing dimer, and extended duplex conformations as previously reported (Figure S1A, lanes 1–3).^14^ Extension of the s2m stem in DIS-s2m resulted in reduced dimer formation (Figure S1A, lanes 4–6). In contrast, the DIS-s2m extended band shows a prominent dimer band (Figure S1A, lanes 7–9). To better understand the dimerization of these constructs, we performed the same experiments but incubated the samples in the presence of increasing Mg^2+^ concentrations at 37 °C for 24 h, conditions which we previously showed promote formation of both kissing dimer and extended duplex conformations (Figure S1B).^14^ As expected, the isolated s2m and DIS-s2m extended constructs exhibited increased dimer formation, with the DIS-s2m still showing reduced dimerization capabilities. Through these experiments and comparison of the secondary structures of these constructs, as well as the known s2m dimerization patterns, we attribute the dimerization of the DIS-s2m extended to the additional 3′-hairpin, which could mediate dimer formation by providing an additional dimerization site (Figure S1C).

**Figure 3:**
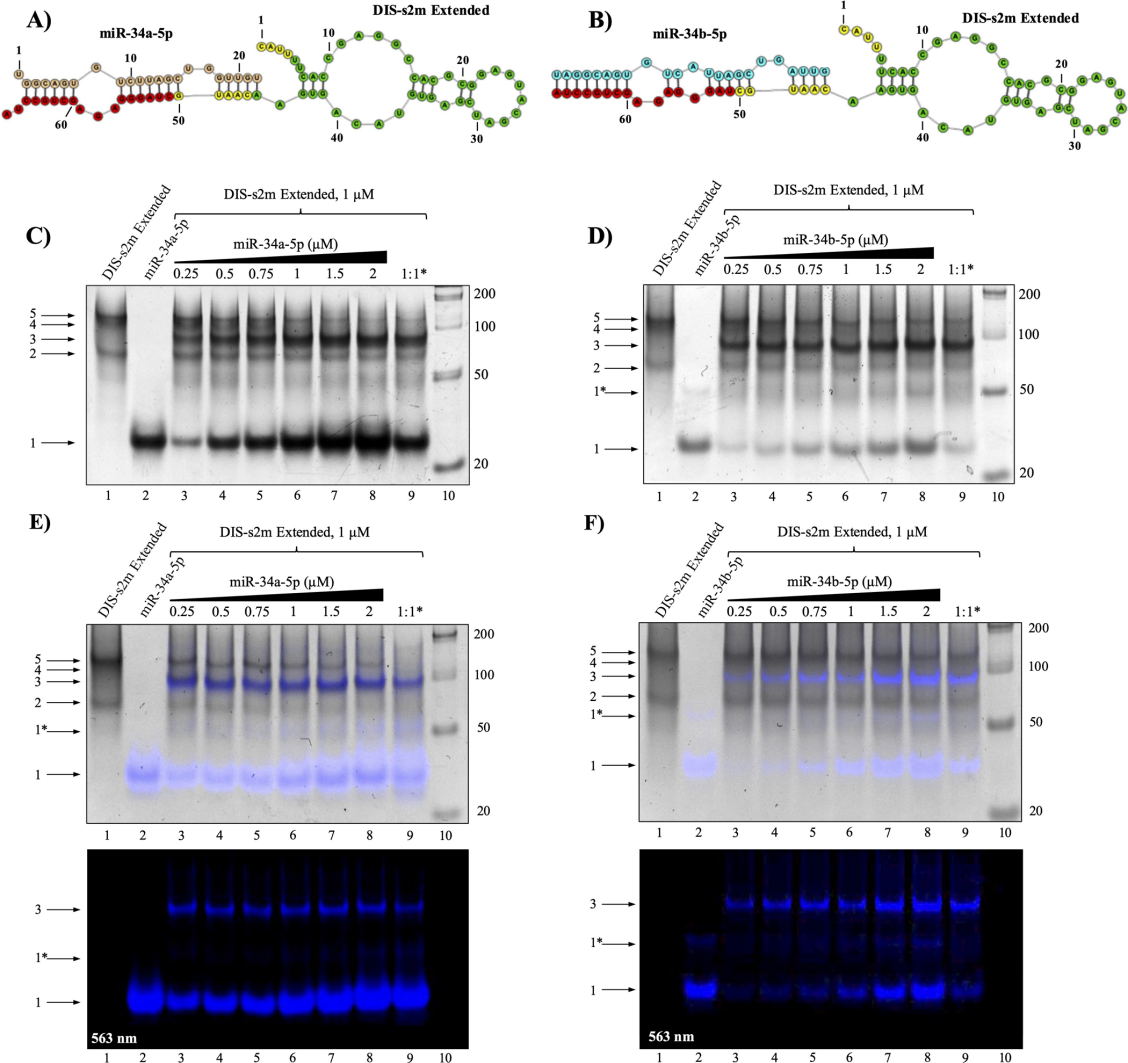
Native PAGE analysis of the binding interactions of miR-34a-5p and miR-34b-5p with DIS-s2m extended. RNAstructure software predicted secondary structures of the 1:1 DIS-s2m extended complex with miR-34a-5p (A) and miR-34b-5p, respectively (B). (C) Native PAGE analysis of miR-34a-5p binding to the DIS-s2m extended peptide demonstrated the formation of a single complex (lanes 3–9, arrow 3). This band is also apparent upon annealing of the two oligomers (lane 9, arrow 3). (D) Native PAGE analysis of miR-34b-5p binding to the DIS-s2m extended revealed the same binding pattern, with the formation of a new band (lanes 3–9, arrow 3) which corresponds to one copy of miR bound to DIS-s2m extended. (E) Native PAGE analysis utilizing a Cy3-tagged miR-34a-5p confirmed the miR-34a-5p complex with DIS-s2m extended as indicated by the fluorescence signature (lanes 3–9, arrow 3), which is observed in both the overlay (top) and the 563 nm filter (bottom) images. Additionally, a higher molecular weight band is observed with fluorescent signature (lanes 2–9, arrow 1*), which we attribute to a dimer of the free miR. (F) Native PAGE analysis utilizing Cy3-miR-34b-5p confirmed the miR-34b-5p complex with DIS-s2m extended (lanes 3–9, arrow 3), shown in both the overlay (top) and the image taken at 563 nm (bottom). Again, we observe a dimer band of the free miR (lanes 2–9, arrow 1*) which is present as increasing miR is titrated.

We used the RNAstructure software to predict the secondary structures of 1:1 complexes for both miR-34a-5p and miR-34b-5p bound to the DIS-s2m extended, and in both cases, the s2m is expected to remain relatively intact (compare Figure 2 and Figures 3A and 3B, green), whereas the small hairpin following it is expected to fully engage in base pairing with the miR (compare Figure 2 and Figures 3A and 3B, red). Next, we performed native PAGE experiments titrating each miR individually to the DIS-s2m extended (Figure 3C and 3D).^49^ As discussed above, the isolated DIS-s2m extended (Figure 3C and 3D, lane 1) exists in equilibrium between monomer (arrow 2) and dimer structures (arrows 4 and 5), whereas miR-34a-5p is mostly monomeric (Figure 3C, lane 2, arrow 1). Upon titration of miR-34a-5p at increasing stoichiometric ratios (Figure 3C, lanes 3–9), a new upper band appears (arrow 3), with a concomitant decrease in the band intensity of both DIS-s2m extended dimer bands (arrows 4 and 5). We assign this new band (arrow 3) to the predicted 1:1 complex of DIS-s2m extended to miR-34a-5p, which is 89 nt (Figure 3A). The decrease in intensity of the DIS-s2m extended dimer bands further supports the idea that the DIS-s2m extended construct forms dimers through dimerization of its 3′-hairpin tail, due to miR binding reducing the potential of the DIS-s2m extended to interact at this site.

Native PAGE experiments for miR-34b-5p binding to the DIS-s2m extended region showed a similar pattern as miR-34a-5p (Figure 3D). A concomitant decrease in DIS-s2m extended dimer bands (Figure 3D, lanes 3–9, arrows 4 and 5) with the appearance of a new band at ∼90 nt (lanes 3–9, arrow 3) indicates the formation of a 1:1 complex of DIS-s2m extended and miR-34b-5p. To confirm these assignments, we repeated the respective binding experiments using Cy3-tagged miR-34a-5p and miR-34b-5p (excitation: 555 nm; emission: 563 nm) (Figure 3E and 3F). We observed a strong Cy3 fluorescence signature that overlays with the previously assigned complex band (Figure 3C and 3D, arrow 3), indicating the presence of the respective miR in the observed complexes. We also noted the appearance of a lower band that migrates at about 50 nt and contains the Cy3 fluorescence signature, which we assign to the dimer of the Cy3-tagged miRs (arrow 1*). In a negative control experiment, we showed that miR-132-3p does not bind to the DIS-s2m extended residue, proving that miR-34a-5p and miR-34b-5p bind specifically (Figure S2).

One dimensional (1D) ^1^H NMR spectroscopy was used to obtain additional information about the binding interactions between miR-34a-5p and miR-34b-5p individually to the DIS-s2m extended, monitoring the changes in the imino proton resonance region 10–15 ppm (Figure S3). Uracil imino protons in U:A base pairs resonate in the 13.0–15.0 ppm range; guanine imino protons in G:C base pairs resonate in the 12.0–13.5 ppm range; and imino protons in G:U base pairs resonate in the 10.0–12.0 ppm range for guanines and 11.0–12.0 range ppm for uracils.^50^ For these experiments, we analyzed samples of the free miR, free DIS-s2m extended, and miR incubated in a 2:1 ratio with DIS-s2m extended. We further analyzed the 2:1 miR:DIS-s2m extended sample by boiling it and slow annealing, conditions that promote forced binding. Upon titration of miR-34a-5p to the DIS-s2m extended and comparison of the miR-bound spectra to the free DIS-s2m extended, we observe six new resonances (13.25 13.09, 12.53, 12.37, 11.23, and 10.98 ppm) indicating the formation of new base pairs (Figure S3A). While we do not have resonance assignments for these spectra, the presence of the resonances at 11.23 and 10.98 ppm indicate the formation of GU base pairs in the complex of miR-34a-5p and DIS-s2m extended. Moreover, many of the resonances originally present in the free DIS-s2m extended were not perturbed, indicating that the predicted s2m structure within the DIS-s2m extended remains intact (Figure 3A, green). While the new resonances indicate the formation of base pairs between DIS-s2m extended and miR-34a-5p, it should be noted that some of the predicted base pairing involves the replacement of intramolecular base pairs of DIS-s2m extended with identical intermolecular base pairs between DIS-s2m extended and miR-34a-5p (compare Figures 2 and 3A), which will likely resonate at similar ppm values. Similarly, we titrated miR-34b-5p to the DIS-s2m extended (Figure S3B) and observed new imino proton resonances between miR-bound and free DIS-s2m extended (13.71 13.31, 13.18, 12.53, 12.39, 11.26, 11.16, and 10.97 ppm), indicating the formation of new base pairs, with no significant loss of original resonances. Taken together, these NMR experiments confirm the formation of the complex between the respective miR and DIS-s2m extended, without major disruption of the base pairing of the s2m element (Figures 3A and 3B, green) within the DIS-s2m extended.

**Figure 4:**
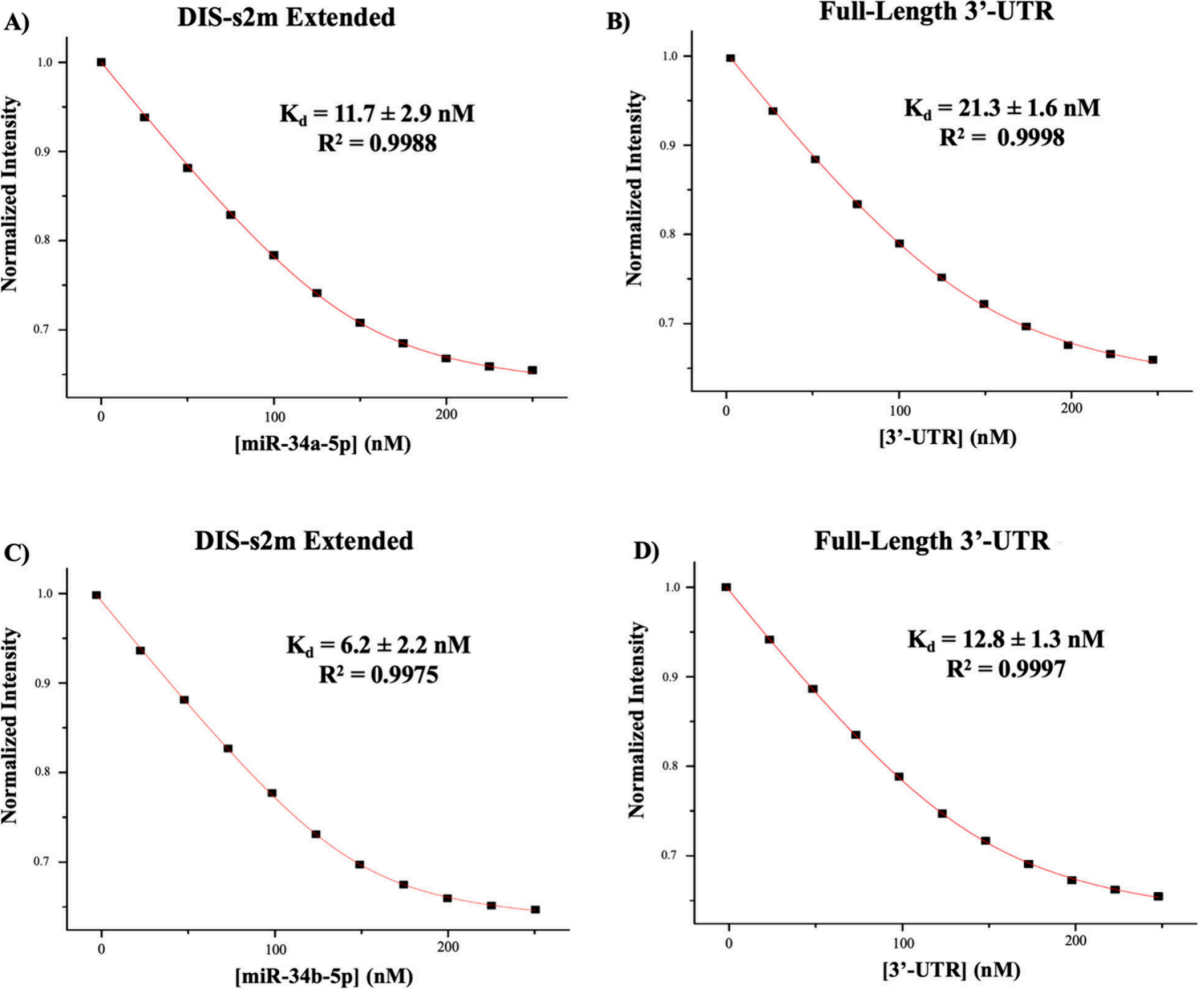
*K*_d_ determination of miR-34a-5p and miR-34b-5p binding to the DIS-s2m extended and the full-length 3′-UTR construct by steady-state fluorescence spectroscopy. (A) The *K*_d_ for the miR-34a-5p:DIS-s2m extended complex was determined to be 11.7 ± 2.9 nM and (B) *K*_d_ for the miR-34a-5p:full-length 3′-UTR complex was determined to be 21.3 ± 1.6 nM. (C) The *K*_d_ for the miR-34b-5p:DIS-s2m extended complex was determined to be 6.2 ± 2.2 nM, and (D) the *K*_d_ for the miR-34b-5p-full-length 3′-UTR complex was determined to be 12.8 ± 1.3 nM.

Next, we used steady-state fluorescence spectroscopy to determine the dissociation constant, *K*_d_, of the complexes formed by each miR with the DIS-s2m extended as well as with the full-length 3′-UTR. We utilized a modified DIS-s2m labeled with pyrollo-cytosine (pyrC, excitation: 350 nm; emission: 445 nm), within the predicted binding site for these miRs on DIS-s2m extended (5′-AGpyrCUGC-3′, bolded in Table 2), titrating the unlabeled miR-34a-5p (Figure 4A) and miR-34b-5p (Figure 4C). The SARS-CoV-2 3′-UTR was obtained by *in vitro* transcription reaction and purified in native conditions to retain its fold.^51^ To measure the miR-34a-5p and miR-34b-5p binding to the full length 3′-UTR, we utilized the Cy3-tagged miRs and titrated the full-length 3′-UTR, monitoring the fluorescence changes upon the complex formation (Figure 4B and 4D). The binding curves for each miR to either DIS-s2m and full-length 3′-UTR were then fit to eq 1 to determine the *K*_d_ of each complex.

For the miR-34a-5p-DIS-s2m extended complex, we determined a *K*_d_ of 11.7 ± 2.9 nM (Figure 4A), whereas for the miR-34a-5p-full length 3′-UTR complex, we determined a *K*_d_ of 21.3 ± 1.6 nM (Figure 4B). Similar experiments were performed for miR-34b-5p, determining a *K*_d_ of 6.2 ± 2.2 nM for its complex with the DIS-s2m extended (Figure 4C) and a *K*_d_ of 12.8 ± 1.3 nM for its complex with the full-length 3′-UTR (Figure 4D). We utilized miR-132-3p as a negative control for the DIS-s2m extended (Figure S4A) and pre-miR-125a for the full-length 3′-UTR and found no significant quenching of the fluorescence intensity (Figure S4B). The *K*_d_ values for the model system binding sites are about half of those measured for the full length 3′-UTR, indicating potential differences in accessibility of the miRs for their binding site when in the context of the full-length 3′-UTR. Nonetheless, this difference in *K*_d_ values results in a difference of only 0.3 kcal/mol in the free energy of binding (Table 1), validating that when in the context of the full-length 3′-UTR these miRs bind to the specific sites we used in the model systems.

**Table 1:** SARS-CoV-2 Model System and Full-Length 3′-UTR Interactions with Host miR-34a-5p, miR-34b-5p, and miR-760-3p Measured by Steady-State Fluorescence Spectroscopy

3'-UTR construct	microRNA/FANA	*K*_d_ (nM)	AG = −*RT* ln(1/*K*_d_) (kcal/mol)
DIS-s2m Extended	miR-34a-5p	11.7 ± 2.9	–10.8 ± 0.1
	miR-34b-5p	6.2 ± 2.2	–11.2 ± 0.2
	FANA-34	8.7 ± 1.1	–11.0 ± 0.1
3′-UTR T100:TL Duplex	miR-760-3p	24.0 ± 4.1	–10.4 ± 0.1
	FANA-760	16.4 ± 1.8	–10.6 ± 0.1
Full-Length 3′-UTR	miR-34a-5p	21.3 ± 1.6	–10.5 ± 0.1
	miR-34b-5p	12.8 ± 1.3	–10.8 ± 0.1
	miR-760-3p	8.8 ± 3.0	–11.0 ± 0.2

Next, we utilized UV thermal denaturation spectroscopy to characterize the stability of the complex formed by each miR with DIS-s2m extended by determining its melting temperature (*T*_m_). For the complex of miR-34a-5p with DIS-s2m extended, we determined a *T*_m_ of 54.0 ± 0.1 °C, and for the complex of miR-34b-5p with DIS-s2m extended, we determined a *T*_m_ of 57.0 ± 0.1 °C (Figure S5A–S5C). These findings indicate that while both complexes are stable at the physiological temperature a slightly more stable complex is formed by miR-34b-5p with DIS-s2m extended as compared with that formed by miR-34a-5p. These results are consistent with the fluorescence spectroscopy results, which show a lower *K*_d_ for the miR-34b-5p-DIS-s2m extended complex as compared with that of the miR-34a-5p-Dis-s2m extended complex.

### Host Cellular miR-760-3p Interacts with the SARS-CoV-2 Genome 3′-UTR Terminus

**Figure 5:**
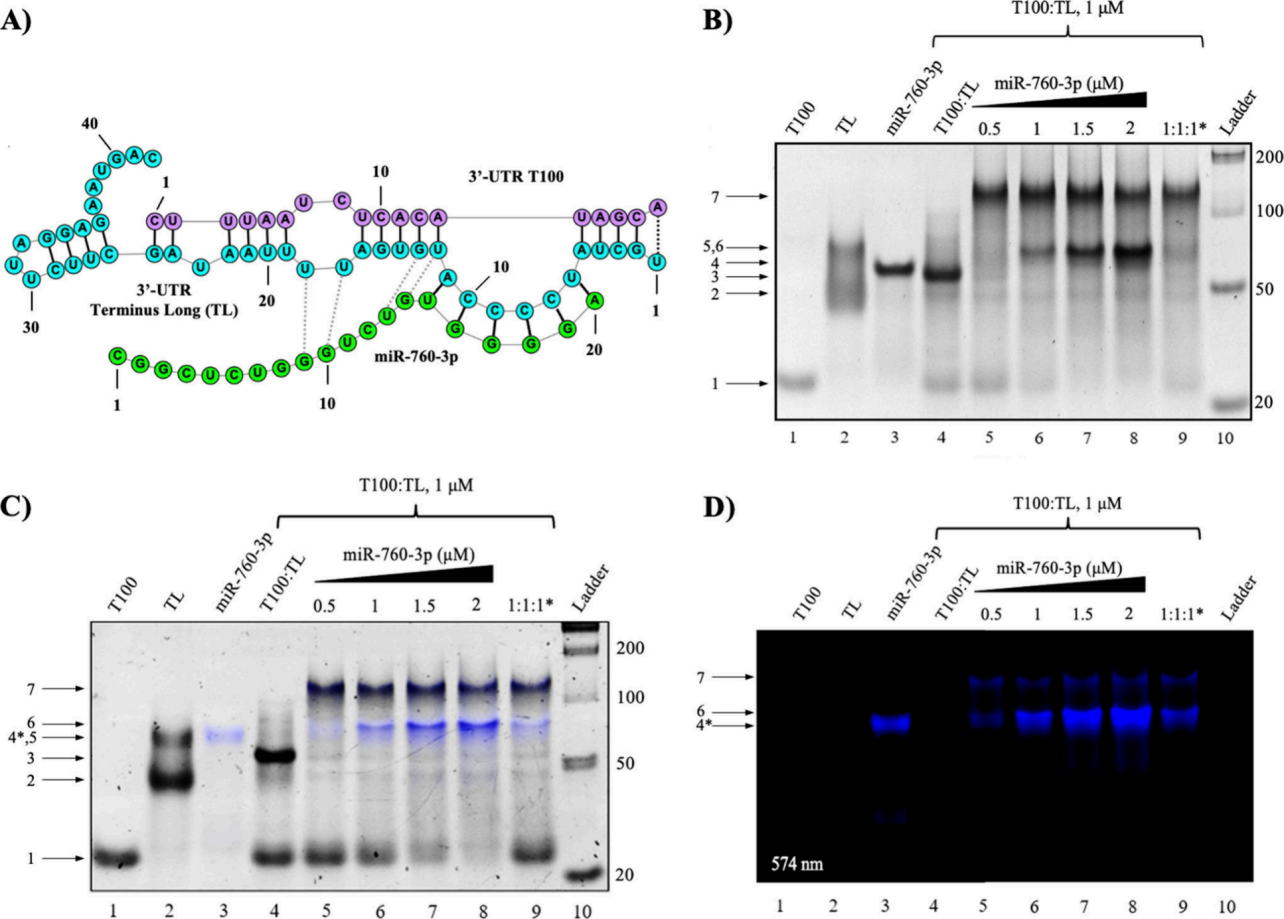
Native PAGE analysis of miR-760-3p binding to the 3′-UTR duplex mimic. (A) The predicted structure of miR-760-3p bound to the preformed T100:TL duplex mimic. Within this complex, we also note the possible formation of additional base pairs between TL and miR-760-3p in the presence of T100 (gray dashed lines). (B) Native PAGE of the miR-760-3p binding to the 3′-UTR T100:TL duplex mimic: two complexes that correspond to a monomer (80 nt) and dimer (160 nt) of miR-760-3p bound to the duplex mimic appear upon the addition of miR-760-3p to the preformed 3′-UTR duplex mimic, in μM ratios (lanes 5–8); in lane 9 labeled by 1:1:1* all three oligomers at 1 μM were slow annealed together. (C) Identical Native PAGE gel of the fluorescently tagged DY547-miR-760-3p binding to the 3′-UTR T100:TL duplex construct confirmed these complexes as containing miR-760-3p, indicated by the DY547 fluorescence signature (lanes 5–9, arrows 6 and 7). (D) The original image was visualized at 545 nm to observe the DY547 fluorescence signature.

We identified that miR-760-3p is predicted to initiate binding at the exposed six-nucleotide bulge located at the SARS-CoV-2 3′-UTR terminus (Figure 2, turquoise dashed line). Thus, to mimic this binding site, we preformed a duplex structure (Figure 5A) using two chemically synthesized sequences (Table 2): the first (nt 102–119 in the 3′-UTR), named here T100 (purple in Figure 2 and 5A), and the second (nt 272–313), named here TL (turquoise in Figures 2 and 5A). We first analyzed the binding of miR-760-3p to its duplex binding site mimic by native PAGE. In a TBM gel, the isolated T100 migrates as a monomer (Figure 5B, lane 1, arrow 1); the TL is present as a mixture of monomer and dimer (Figure 5B, lane 2, arrows 2 and 5); and miR-760-3p forms a higher molecular weight complex that based on its migration is likely a trimer (Figure 5B, lane 3, arrow 4). When slow annealed together, TL forms a stable duplex with T100 (at ∼60 nt; Figure 5B, lane 4, arrow 3). Upon the addition of increasing concentrations of miR-760-3p, this complex band disappears with the concomitant appearance of two new upper bands, which we attribute to the complex between the 3′-UTR T100:TL duplex and one copy of miR-760-3p (∼80 nt) (Figure 5B, arrow 6) and to the dimer of this complex, respectively (Figure 5B, arrow 7).

To confirm the presence of miR-760-3p in these higher molecular complexes, we labeled it with a DY547 fluorophore at its 5′ end (DY547-miR-760-3p) and performed similar TBM PAGE binding experiments. The gels were visualized by monitoring the DY547 fluorescence signal (excitation: 558 nm; emission: 574 nm), followed by SYBR gold staining (Figure 5C and 5D). Of note, DY547-miR-760-3p showed altered migration patterns (Figure 5C and 5D, lane 3, arrow 4*). The two upper bands previously assigned to the 3′-UTR T100:TL-miR-760-3p complex (arrow 6) and to its dimer (arrow 7) have the fluorescence signature of DY547 as seen in an overlay of the images of the gel visualized by the DY547 fluorescence and by the SYBR gold stain (Figure 5C, lanes 5–9, arrows 6 and 7), confirming the presence of miR-760-3p in these complexes. These results indicate that miR-760-3p does not displace T100 from the preformed T100:TL duplex, suggesting that it initiates binding at the exposed UCCCCA bulge as predicted (Figure 5A). To confirm that miR-760-3p is binding to the predicted exposed bulge, we mutated the TL sequence at that site (Figure 5A, nt 24–29, bolded in Table 2), to produce the 3′-UTR TL mutant and showed that this mutation abolished the binding of miR-760-3p (Figure S6). Further PAGE experiments demonstrated this binding site is specific for miR-760-3p as neither miR-34a-5p nor miR-1307-3p, which were used as negative controls, bound to the 3′-UTR T100:TL duplex (Figure S7).

To confirm that the T100:TL duplex remains intact upon miR-760-3p binding, we performed 1D ^1^H NMR spectroscopy (Figure S8). Upon addition of miR-760-3p to the 3′-UTR T100:TL in a 2:1 ratio, we observed seven new resonances (14.04, 13.53, 12.57, 12.22, 11.79, 11.70, and 11.43 ppm) as compared to the spectrum of the free 3′-UTR T100:TL duplex, indicating the formation of new base pairs in the complex. We also noted that most of the resonances present in the T100:TL duplex remain unchanged, confirming that the binding of miR-760-3p does not displace T100 from the T100:TL duplex. However, while we do not have specific assignments for these imino proton resonances, the presence of the resonances at 11.79, 11.70, and 11.43 ppm indicates the formation of GU base pairs in the T100:TL duplex-miR-760-3p complex, suggesting that besides the base pairs formed at the exposed bulge in the T100:TL duplex additional GU base pairs are formed with miR-760-3p (Figure 5A, dashed lines). Taken together, these experiments show that host miR-760-3p binds to its predicted binding site without disruption of the original 3′-UTR T100:TL duplex mimic.

**Figure 6:**
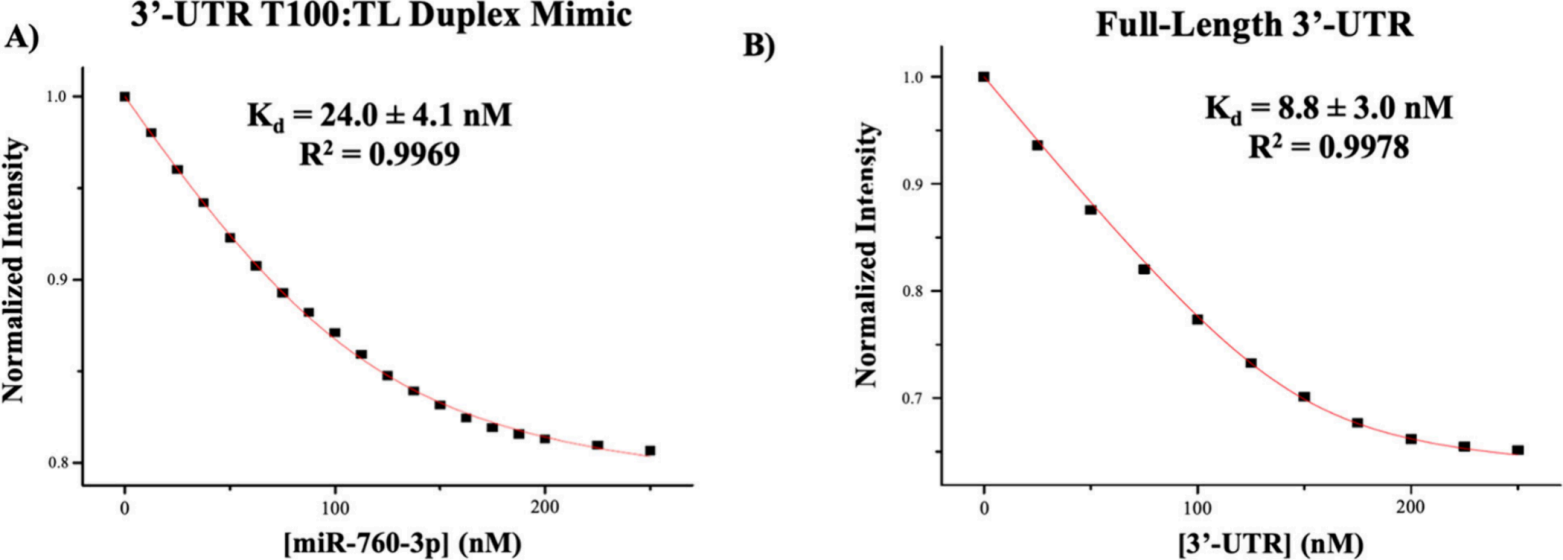
Determination of *K*_d_ of miR-760-3p binding to the 3′-UTR T100:TL duplex mimic and to the full-length 3′-UTR by steady-state fluorescence spectroscopy. (A) The *K*_d_ for the miR-760-3p:3′-UTR T100:TL duplex mimic was determined to be 24.0 ± 4.1 nM, and (B) the *K*_d_ for the miR-760-3p:full-length 3′-UTR complex was determined to be 8.8 ± 3.0 nM.

Quantitative analysis of miR-760-3p binding to the 3′-UTR T100:TL duplex was obtained by steady-state fluorescence spectroscopy (Figure 6). A 3′-UTR TL sequence containing a pyrC in the exposed bulge of the miR-760-3p binding site (5′-UCpyrCCCA-3′, bolded in Table 2) was used to preform the 3′-UTR T100:TL duplex mimic, followed by titration of the wild-type miR-760-3p. The binding curve (Figure 6A) was fitted with eq 1 to determine the dissociation constant, *K*_d_, of 24.0 ± 4.1 nM for the miR-760-3p-3′-UTR T100:TL complex. In control experiments, we titrated miR-34a-5p to the pyrC-tagged 3′-UTR T100:TL duplex mimic and observed no quenching of the fluorescence signal (Figure S8A). Interestingly, the predicted free energy of binding of miR-760-3p to only the UCCCCA bulge of TL, calculated using the RNA structure software, is −10.2 kcal/mol, which is very close to the free energy of its binding to the T100:TL duplex of −10.4 ± 0.1 kcal/mol determined experimentally by fluorescence spectroscopy (Table 1). This is consistent with our native PAGE and 1D NMR results which indicate that the miR-760-3p binding to the T100:TL duplex does not require the displacement of the T100 sequence.

**Table 2:** Selected Oligonucleotides Used in This Study^a^

Sequence Name	Sequence (5′-3′)
**3′-UTR T100**	5′-CUUUAAUCUCACAUAGCA-3′
**3′-UTR Terminus Long (TL)**	5′-UGCUAUC**C**CCAUGTGAUUUUAAUAGCUUCUUAGGAGAAUGAC-3′
**3′-UTR TL Mutant**	5′-UGCUA**ACACA**AUGUGAUUUUAAUAGCUUCUUAGGAGAAUGAC-3′
**DiS-s2m Extended**	5′-CAUUUUCACCGAGGCCACGCGGAGUACGAUCGAGUGUACAGUGAACAAUGCUAGGGAGAG**C**UGCCUA-3′
**miR-760-3p**	5′-CGGCUCUGGGUCUGUGGGGA-3′
**miR-34a-5p**	5′ -UGGCAGUGUCUUAGCUGGUUGU-3′
**miR-34b-5p**	5′-UAGGCAGUGUCAUUAGCUGAUUG-3′
**FANA-760**	5′-AAGCUAUUAAAAUCACAUGGGGA-3′
**FANA-34**	5′-UAGGCAGCUCUCCCUAGCAUUGU-3′
**SARS-**CoV-2 Genome 3′-UTR	5′-AUGGGCUAUAUAAACGUUUUCGCUUUUCCGUUUACGAUAUAUAGUCUACUCUUGUGCAGAAUGAAUUCUCGUAACUACAUAGCACAAGUAGAUGUAGUUAACUUUAAUCUCACAUAGCAAUCUUUAAUCAGUGUGUAACAUUAGGGAGGACUUGAAAGAGCCACCACAUUUUCACCGAGGCCACGCGGAGUACGAUCGAGUGUACAGUGAACAAUGCUAGGGAGAGCUGCCUAUAUGGAAGAGCCCUAAUGUGUAAAAUUAAUUUUAGUAGUGCUAUCCCCAUGUGAUUUUAAUAGCUUCUUAGGAGAAUGAC-3′
**miR-132-3p**	5′-UAACAGUCUACAGCCAUGGUCG-3′
**miR-1307-3p**	5′-ACUCGGCGUGGCGUCGGUCGUG-3′
**pre-miR-125a**	5′-UGCCAGUCUCUAGGUCCCUGAGACCCUUUAACCUGUGAGGACAUCCAGGGUCACAGGUGAGGUUUUGGGAGCCUGGCGUCUGGCC-3′

a Sequences that contain mutations or are pyrollo-cytosine-labeled are indicated with the point mutations in bold.

Next, we assessed the binding of miR-760-3p to the full-length SARS-CoV-2 3′-UTR using DY547-miR-760-3p and monitoring its fluorescence quenching upon binding to the unlabeled full-length 3′-UTR (Figure 6B).^51^ The experiments were performed in triplicate, determining a *K*_d_ of 8.8 ± 3.0 nM for the DY547-miR-760-3p-3′-UTR complex. Pre-miR-125a was titrated to the DY547-miR-760-3p as a negative binding control for the full-length 3′-UTR, and again, no significant quenching of the signal was observed (Figure S8B). MiR-760-3p binds tighter to the full-length 3′-UTR than to the T100:TL duplex, which could be due to a difference in accessibility of its binding site in the T100:TL duplex as compared with the full-length 3′-UTR (Figure 2 and Figure 5A, turquoise). When in the context of the T100:TL duplex, the TL sequence can fold into a short hairpin at its 3′-end using the sequence CUUCUUAGGAG (Figure 5A, turquoise) or use the same nucleotides to dimerize at its 3′-end. This folding could reduce the accessibility of miR-760-3p for its binding site as compared to the context of the full length 3′-UTR where this sequence is engaged in base pairs with sequences upstream of the PK stem-loop (Figure 2, turquoise).^52^ Nonetheless, this difference in *K*_d_ values results in a difference of only 0.5 kcal/mol in the free energy of these binding interactions (Table 1).

Additional characterization of the miR-760-3p binding interactions was performed using UV thermal denaturation spectroscopy, in which the melting temperature of the miR-760-3p-3′-UTR TL complex was determined to be 52.0 ± 0.1 °C, showing that it is thermodynamically stable at the physiological temperature (Figure S5A, S5D). Taken together, our results show that all three miRs investigated form stable complexes with their predicted binding sites, both in model systems and in the full-length SARS-CoV-2 3′-UTR. The *K*_d_ values for the model systems and the full length 3′-UTR are comparable to the *K*_d_ values measured for the *in vitro* binding of miR-122 (in the absence of AGO2) to the HCV 5′-UTR site 1 (11.1 ± 1.5 nM) but significantly lower than the *K*_d_ measured for miR-122 binding to THE HCV 5′-UTR site 2 (979 ± 84 nM).^53^ AGO2 enhances the binding of miRs to their targets; for example, the *K*_d_ values were lower by at least 100-fold for the miR-122 binding in the presence of AGO2 to the HCV 5′-UTR sites 1 and 2, respectively.^54^

Thus, we expect that the *K*_d_ values measured here will be lower by at least 100-fold if the miRs binding to the SARS-CoV-2 3′-UTR is assisted by AGO2. Interestingly, however, it has been shown that in HeLa cells 30%–90% of miRs are not bound by AGO2 and, moreover, that preformed miRNA–mRNA duplexes exist endogenously, in a 7-fold excess of miR relative to AGO 1–4 bound to mRNA.^55^ Thus, while our experiments were performed *in vitro*, in the absence of the AGO 1–4 proteins, it is feasible that, similarly to their mRNA targets, the SARS-CoV-2 3′-UTR can also preform duplexes with these miRs *in vivo*, not requiring the assistance of AGO2.

A global analysis of human-infecting viruses has identified that they are more likely to contain human miR binding sites and that these binding sites are particularly enriched in ssRNA viruses.^56^ We speculate that by binding these miRs the SARS-CoV-2 3′-UTR which is present on both the genomic and subgenomic viral RNA can act as sponges, preventing them from exerting their normal cellular function (Figure 1B). The probability of the SARS-CoV-2 viral genome 3′-UTR competing with the mRNA targets of these miRs through sponging them is high, given the estimated 10^9^–10^11^ copies of viral RNA present in infected lung tissues.^57^ This miR “sponging” effect has also been previously demonstrated in other viral systems. The Epstein–Barr virus, albeit containing a double-stranded DNA viral genome, produces circular RNAs which were found to “sponge” host miRs involved in a plethora of pathways, most notably in the interferon response and immune cell activation signaling cascades, suppressing innate immunity to favor viral replication.^58,59^ In the case of the hepatitis C virus (HCV), the binding of miR-122 to the genome’s 5′-UTR induces a secondary structure change which enhances binding of ribosomes to the internal ribosomal entry site, which has been found to favor the viral life cycle by enhancing viral protein translation.^60−62^ Notably, the binding of miR-122 to the HCV viral genome does not act like the normal miR function of translation inhibition. HCV is not the only virus which exhibits this, as many other RNA viruses are particularly adept to evading the natural inhibitory function of miRs.^60,61,63^ The eastern equine encephalitis virus (EEEV) has been demonstrated to bind miR-142-3p, this interaction allowing the virus to suppress the innate immune response by disrupting the cytokine production. In another case, the neuron-specific miR-138 is demonstrated to bind to the herpes simplex virus-1 viral genome 3′-UTR to promote viral latency and reduce immune activation.^64^ As discussed earlier, Scheel et al. demonstrated that pestiviruses utilize host miRs to assist in the viral life cycle, as they propose that miR-17 binding exerts a protective effect on the viral genome 3′-UTR by binding and reducing degradation.^15^ Moreover, the host miR-10 passenger strand (miR-10*) binds to the coxsackie virus genome coding regions and is shown to directly promote viral translation.^65^ Thus, it is not uncommon for viruses, particularly RNA viruses, to hijack host miRs for the benefit of the viral life cycle, as we propose here.

However, further *in vivo* experiments are required to validate this “sponging” model, and as based on the data presented here we cannot exclude the possibility that the miRs binding by the SARS-CoV-2 3′-UTR could confer increased stability to the viral genome or assist in the virus replication and/or translation, as observed in some of the other viral systems discussed above.

### FANA-760 and FANA-34 as Competitive Inhibitors for the miR-34a-5p, miR-34b-5b, and miR-760-3p Binding to the SARS-CoV-2 3′-UTR

**Figure 7:**
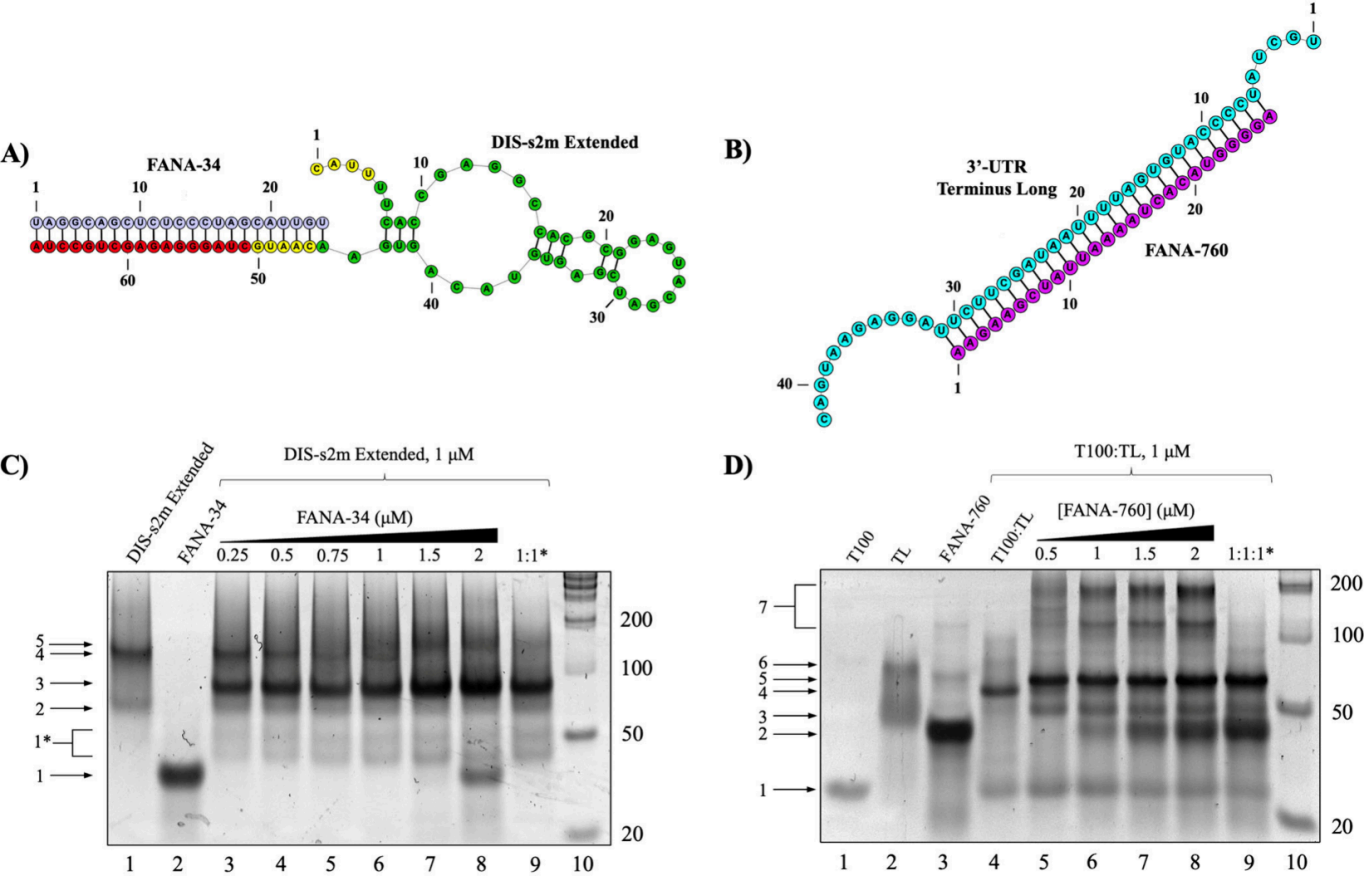
Native PAGE of the FANA-760 and FANA-34 oligonucleotides binding to the 3′-UTR duplex mimic and the DIS-s2m extended, respectively. Both FANA-34 and FANA-760 are designed as perfect complements to their respective binding site, as shown through the predicted structures of (A) FANA-34 bound to the DIS-s2m extended and (B) FANA-760 bound to the 3′-UTR duplex mimic. (C) Native PAGE analysis: a complex band between FANA-34 and DIS-s2m extended forms upon the addition of FANA-34 stoichiometrically (lanes 3–9, arrow 3). (D) Native PAGE analysis shows the FANA-760 predominantly forming a 1:1 duplex with the TL (lanes 5–9, arrow 5).

Next, we designed antisense oligonucleotides (ASOs) that target the miR-34a-5p, miR-34b-5p, and miR-760-3p binding sites on the SARS-CoV-2 3′-UTR. Nucleic acid analogs have been employed as therapeutic agents in many disorders, allowing for sequence specific interactions with genomic or proteomic targets.^66−69^ Of particular interest in this study, we focused on 2′-fluoro-d-arabinonucleic acids (FANAs), as these oligomers have been shown to be self-delivering *in vivo* and provide effective binding to their targets both in *in vitro* and *in vivo* systems.^68−70^ We designed the FANA-34 oligomer (Table 2) as a perfect complement to the predicted miR-34a/b-5p binding site (Figure 7A) and analyzed its binding to DIS-s2m extended by native PAGE (Figure 7C). FANA-34 migrates as a monomer (Figure 7C, lane 2, arrow 1) and forms a 1:1 complex with DIS-s2m extended (91 nt; Figure 7C, lanes 3–9, arrow 3). Higher molecular weight bands are also present (Figure 7C, lanes 6–8, arrow 5), which we attribute to a dimer of the FANA-34-DIS-s2m extended. A concomitant decrease in intensity of both DIS-s2m extended dimer structures, which migrate as a single band, is observed (Figure 7C, lanes 1 and 3–9, arrow 4). With additions of FANA-34, two lower molecular weight bands appear (Figure 7C, lanes 3–9, arrow 1*), which we attribute to degradation products of the DIS-s2m extended forming complexes with FANA-34.

We designed FANA-760 (Table 2) as a perfect complement to the miR-760-3p predicted binding site on the 3′-UTR TL sequence (Figure 7B) and analyzed its binding by native PAGE experiments (Figure 7D). FANA-760 migrates primarily as a dimer (Figure 7D, lane 3, arrow 2) with several higher molecular weight complexes also being present. Upon addition of the FANA-760 to the preformed 3′-UTR T100:TL duplex, a prominent new band is apparent which we assign to the TL:FANA-760 complex (68 nt) (Figure 7D, lanes 5–9, arrow 5), concomitant with the disappearance of the band corresponding to the 3′-UTR T100:TL duplex (Figure 7D, lanes 5–9, arrow 4). Higher molecular weight complexes are also observed (Figure 7D, lanes 5–8, bracket 7), which could originate from a dimer and trimer of the TL:FANA-760 complex (Figure 7D, lanes 5–8, bracket 7), as the TL sequence is capable of dimerizing at its 3′ end as well as at its 5′ end. Interestingly, these higher molecular weight complexes disappear when TL, T100, and FANA-760 are slow annealed together (Figure 7D, lane 9). These results show that the FANA-760 oligomer is able to displace the T100 sequence from the preformed 3′-UTR T100:TL duplex.

**Figure 8:**
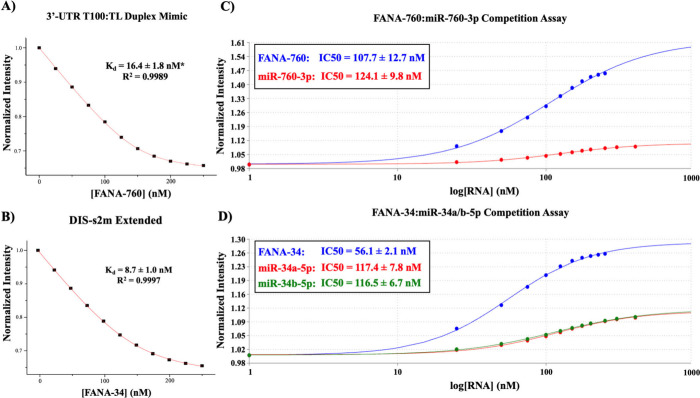
Determination of FANA-760 and FANA-34 binding interactions to the SARS-CoV-2 3′-UTR by steady-state fluorescence spectroscopy. (A) The *K*_d_ for FANA-760 to the 3′-UTR T100:TL duplex complex was determined to be 16.4 ± 1.8 nM. (B) The *K*_d_ value for the FANA-34:DIS-s2m extended was determined to be 8.7 ± 1.0 nM. (C) Competition assays allowed the determination of IC50 values of 107.7 ± 12.7 nM for FANA-760 and 124.1 ± 9.8 nM for miR-760-3p binding to the full-length 3′-UTR and (D) IC50 values of 56.1 ± 2.1 nM for FANA-34, 117.4 ± 7.8 nM for miR-34a-5p, and 116.5 ± 6.7 nM for miR-34b-5p binding to the full-length 3′-UTR.

To obtain quantitative information about the binding of FANA-760 and FANA-34 to the SARS-CoV-2 genome 3′-UTR, we performed steady-state fluorescence spectroscopy utilizing the pyrC-tagged 3′-UTR T100:TL duplex and DIS-s2m extended sequences used in the wild-type miRNA steady-state fluorescence spectroscopy experiments (Figure 8A and 8B). FANA-760 was titrated to the 3′-UTR T100:TL duplex, and FANA-34 was titrated to the DIS-s2m extended sequences, respectively, in a similar manner to prior experimentation, after which each binding curve was fit to eq 1 to determine the *K*_d_ for each complex. We determined a *K*_d_ of 16.4 ± 1.8 nM for the complex formed by FANA-760 with the 3′-UTR T100:TL duplex mimic (Figure 8A) and a *K*_d_ of 8.7 ± 1.0 nM for the complex formed by FANA-34 with the DIS-s2m extended (Figure 8B). These *K*_d_ values are comparable with those measured for the respective miRs binding to the same binding sites (Table 1) and also comparable to pharmacologically relevant *K*_d_ values measured for small molecules, antibodies, and RNA therapeutics alike.^71^ miR-34a-5p and miR-34b-5p are predicted to have extensive base pairing with their binding sites within the DIS-s2m extended (Figures 3A and 3B), so it is not surprising that their free energy of binding is comparable to that of the FANA-34 (Table 1) which is designed to be fully complementary to the same binding site (Figure 7A). However, the findings that the free energies of binding of miR-760-3p and FANA-760 for the TL-T100 duplex are comparable suggest that even upon displacing the T100 sequence from the duplex FANA-760 does not form a perfect duplex with TL, despite being designed to have full complementarity (Figure 7B). This could be due to the folding of the 3′-end of TL sequence CUUCUUAGGAG into a hairpin or due to its dimerization which prevents the formation of base pairs with its complementary FANA-760 sequence.

Next, to determine if the FANAs are able to compete with their respective miR, we performed competition assays, calculating the IC50 and *K*_I_ for each FANA. In these assays, we incubated the Cy3- or DY547-tagged miRNAs with the full-length 3′-UTR and titrated the respective FANA, monitoring the increase in the fluorescence intensity as the miRs are outcompeted by their respective FANA. The experiments were repeated by titrating unlabeled miR-760-3p or miR-34a/b-5p and monitoring the displacement of the fluorescently tagged miRs. The fluorescence intensities in all competition assays were normalized to the first data point prior to titration of the unlabeled ligand (FANA or miR) and fit with eq 2 to determine the IC50 (Figure 8C and 8D). The IC50 values were then used in eq 3 to determine the *K*_I_ values. For FANA-760, we determined an IC50 of 107.7 ± 12.7 nM, whereas for miR-760-3p we determined an IC50 of 124.1 ± 9.8 nM (Figure 8C). The *K*_I_ of FANA-760 was calculated to be 6.0 ± 0.2 nM. For FANA-34 we determined an IC50 of 56.1 ± 2.1 nM, while miR-34a-5p was determined to have an IC50 of 117.4 ± 7.8 nM and miR-34b-5p had an IC50 of 116.5 ± 6.7 nM (Figure 8D). Using these IC50 values, a *K*_I_ of 7.0 ± 1.9 was calculated for FANA-34 to the full-length 3′-UTR (Figure 8D). These IC50 values, along with the respective *K*_I_ for the FANAs, are physiologically relevant and plausible for implementation in live organisms.^72,73^ In the model system binding experiments, we determined that the binding affinities of both FANA-760 and FANA-34 are comparable to those of miR-760-3p, miR-34a-5p, and miR-34b-5p, respectively (Table 1). In the case of the full-length 3′-UTR, we cannot directly compare the binding affinities of FANA-34 and FANA-760 with those of the miRs. However, the results of the competition experiments showing comparable IC50 values between FANA-760 and miR-760-3p suggest a similar binding affinity of these oligomers to the full-length 3′-UTR. In contrast, FANA-34 IC50 is almost half of the IC50 of miR-34a-5p or miR-34b-5p, suggesting that is has a higher binding affinity for the full-length 3′-UTR than the miRs. This could be due to the fact that within the full-length 3′-UTR the miR-760-3p binding site is located at the end of the genome and more easily accessible, whereas that of miR-34a-5p/miR-34b-5p is in the middle of the 3′-UTR, hindering its accessibility as compared to the model system DIS-s2m extended. Thus, the fact that FANA-34 is perfectly complementary to the binding site might contribute to its increased binding affinity in the context of the full-length UTR. As a negative control, we titrated miR-132-3p instead of FANA-760 and FANA-34, revealing no increase in fluorescence intensity (Figure S9), supporting the ability of the FANA oligomers to compete with the wild-type miRs for binding to the full-length 3′-UTR and confirming that the interactions are sequence specific.

Given the ability of the FANAs to compete with the miRs for their respective binding sites on the SARS-CoV-2 3′-UTR, we propose that the FANA analogs of these miRs could be developed into effective therapeutics for SARS-CoV-2. Reports of severe immune dysregulation upon SARS-CoV-2 infection have accumulated, all centered around hyperactivity of the immune response.^18,22,34,74−80^ Review of clinical data and -omics analysis of SARS-CoV-2 infected patients has suggested a large population of hyperinflammatory phenotypes and further correlates these phenotypes with the onset of ARDS.^81−83^

While the exact cause of ARDS can vary across pathologies, immune dysregulation and hyperexpression of central cytokines, such as IL-6, remain focal points.^84^ A multitude of signaling pathways are affected by SARS-CoV-2 infection, including the JAK/STAT signaling pathway, which is considered to play an essential role due to its activation by various ILs and propagation of the immune response.^19,85−87^ The JAK/STAT pathway is overactive in SARS-CoV-2 infected cells, which is particularly interesting given that ACE2 expression increases upon activation of JAK/STAT.^40,42^ It is known that each of the miRs in this study regulates key biomolecules in the activation of JAK/STAT, and as such, we speculate that this dysregulation may be due to miR “sponging” by SARS-CoV-2. Although the recent variants of SARS-CoV-2 are less severe than the original Wuhan strain, severe symptoms associated with SARS-CoV-2 infection still pose a danger to those that are immunosuppressed or compromised.^82,83,88^ Thus, FANAs or other ASOs that disrupt the miR binding interactions with the SARS-CoV-2 3′-UTR could be developed into potential therapeutics to restore JAK/STAT regulation by PAI-1, GRN, and IL-6.

## Conclusions

We characterized here the binding interactions of miR-34a-5p, miR-34b-5p, and miR-760-3p to their predicted binding sites within the SARS-CoV-2 viral genome 3′-UTR and propose that these interactions prevent these miRs from regulating the translation of key molecules implicated in the JAK/STAT pathway. Clinical data supporting the dysregulation of GRN, PAI-1, IL-6, IL-6R, and GRN in patients of COVID-19 emphasize the potential role of these binding interactions in disease severity while also highlighting a niche therapeutic strategy toward managing severe infections.^18,22,22^ Thus, our results that FANA-760 and FANA-34 can act as competitive inhibitors of the respective miRs binding to SARS-CoV-2 3′-UTR highlight their potential as therapeutic agents.

## Methods

### Oligonucleotide Synthesis

The miR-34a-5p, miR-34b-5p, and miR-760-3p binding sites were based on the predicted fold of the SARS-CoV-2 genome 3′-UTR by RNAstructure.^49^ All RNAs (Dharmacon, Inc.) and FANA oligomers (AUM Biosciences, LLC) used in this study (Table 2) were chemically synthesized and were resuspended in sterile cacodylic acid (10 mM, pH 6.5).

The full-length SARS-CoV-2 genome 3′-UTR was transcribed in native conditions from a psp64 dsDNA plasmid (a kind gift from Dr. Anna Wacker, Institute for Organic Chemistry and Chemical Biology, Germany), as described previously.^51^

### Native Polyacrylamide Gel Electrophoresis

In the dimerization gel comparing the DIS-s2m extended, DIS-s2m, and the isolated s2m, samples of each oligomer (at 1 μM) were snap cooled on dry ice for 5 min, followed by equilibration to 23 °C on the benchtop. After incubating them for an additional hour on the benchtop with 1 mM, 5 mM, and 10 mM MgCl_2_, the samples were electrophoresed on 15% TBM gels at 75 V and 4 °C for 4 h, followed by visualization using SYBR gold stain. A similar experiment was performed, incubating with MgCl_2_ at 37 °C for 24 h.^14^

DIS-s2m extended, miR-34a-5p, and miR-34b-5p samples were snap cooled on dry ice for 5 min, followed by equilibration to 23 °C on the benchtop. Either miR-34a-5p or miR-34b-5p was added to the DIS-s2m extended (at 1 μM), in increasing concentrations (0.25 μM, 0.5 μM, 0.75 μM, 1 μM, 1.5 μM, and 2 μM) with MgCl_2_ (1 mM), and incubated for 1 h at 23 °C. These samples were electrophoresed on 15% acrylamide:bisacrylamide tris-boric acid with 5 mM MgCl_2_ (TBM) gels at 75 V and 4 °C for 4 h visualizing the gels using SYBR gold stain. A negative control for the DIS-s2m extended binding experiments was performed using miR-132-3p, previously used as a negative binding control for native PAGE experiments of the isolated s2m.^14^

To test the binding of miR-760-3p to the SARS-CoV-2 3′-UTR, we utilized a model system of the binding site comprised of the T100 and TL sequences (Figure 2, Table 2) which were boiled together and slow annealed for 1 h. The free T100 and TL sequences were boiled for 5 min and snap-cooled on dry ice. MgCl_2_ (1 mM) was added to the 3′-UTR T100:TL duplex mimic (at 1 μM), along with increasing additions of miR-760-3p (which was previously boiled and snap-cooled): 0.5 μM, 1 μM, 1.5 μM, 2 μM. An additional control was prepared with all three sequences (T100, TL, and miR-760-3p, 1 μM) slow annealed together to promote binding of miR-760-3p to the T100:TL duplex mimic. All samples were incubated at 23 °C for 1 h, and electrophoresed on 12% acrylamide:bisacrylamide TBM gels run at 75 V for 4 h in 1/2x TBM buffer at 4 °C, after which the gel was stained in SYBR gold.^89^ The gels were visualized at 302 nm on a ProteinSimple AlphaImager HP. The negative binding control experiments utilizing miR-34a-5p and miR-1307-3p, along with the binding experiments with the TL mutant sequence, were repeated following the exact procedure described above for miR-760-3p PAGE experiments.

For the experiments using Cy3-miR-34a-5p, Cy3-miR-34b-5p, and DY547-miR-760-3p, the exact procedures were repeated, this time using the labeled miRs. The fluorescent signatures of the DY547 and Cy3 tags were visualized using a broad excitation 600 nm filter, revealing only the tagged RNAs. The gel was then visualized using SYBR gold staining and overlaid with the fluorescent image.

The FANA-34 and FANA-760 oligomers were prepared in the exact procedure as described for the prior native PAGE experiments. The FANA-34 and FANA-760 were incubated with their respective RNA targets and electrophoresed with the same native PAGE procedure as described above, with the gels being stained in SYBR gold. All native PAGE binding experiments for all miRs and FANAs were performed in triplicate.

### Steady-State Fluorescence Spectroscopy

Steady-state fluorescence spectroscopy experiments were performed at 25 °C on a Horiba Jobin Yvon Fluoromax-4C instrument with accompanying software, fitted with a 150 W ozone-free xenon arc lamp. The experiments were performed using a 3 mm path-length quartz cuvette (Starna cells) with the samples being 150 μL. The excitation wavelength was set to 350 nm for pyrollo-cytosine (pyrC) and emission data was acquired from 400 to 500 nm.

The sample was prepared using the pyrC tagged DIS-s2m extended (pyrC-DIS-s2m extended, pyrC is bolded in Table 2) at 150 nM in 10 mM cacodylic acid, pH 6.5, boiled and snap cooled as described above, followed by incubation with MgCl_2_ (1 mM) for 1 h. Snap-cooled miR-34a-5p or miR-34b-5p was titrated in 25 nM increments to a final concentration of 250 nM. Upon each addition, the sample was allowed to equilibrate for 15 min prior to measuring the emission intensity at 445 nm. These experiments were repeated in triplicate for both miR-34a-5p and miR-34b-5p, and the data was fit to eq 1 to determine the dissociation constant K_d_. In these experiments, [X] is the concentration of either miR and [Y] is the concentration of the pyrC-DIS-s2m extended. For a negative control, this experiment was repeated using miR-132-3p. For analysis of miR-760-3p binding, 3′-UTR T100 and pyrC-labeled 3′-UTR TL (pyrC-TL, pyrC is bolded in Table 2) at 150 nM were boiled and slow annealed to preform the 3′-UTR duplex mimic. miR-760-3p was titrated in 12.5 nM increments to a final concentration of 250 nM, equilibrating the sample for 15 min between additions. These experiments were repeated in triplicate, and the collected data was normalized and fit to eq 1. In this case, [X] is the concentration of the titrated miR-760–3p, and [Y] is the concentration of the 3′-UTR duplex mimic. For negative binding control of miR-760–3p binding experiments, we used miR-34a-5p.

To assess binding of the miR-34a-5p, miR-34b-5p, and miR-760-3p to the full length 3′-UTR, a modified version of the previously described experiments was utilized. The Cy3 (miR-34a-5p and miR-34b-5p) and DY547 (miR-760-3p) tagged miRs were prepared at a final concentration of 150 nM. For the Cy3 fluorophore, the excitation wavelength was set to 550 nm, with the emission recorded at 563 nm. For the DY547 fluorophore, the excitation wavelength was set to 558 nm, and the emission was recorded at 574 nm. The full length 3′-UTR was titrated in 25 nM increments to the tagged miRs, and the normalized binding curves for each miR were fit to eq 1, where in this case [X] is the 3′-UTR concentration and [Y] is the concentration of the Cy3/DY547-miRs. To establish specificity of the miRs for the SARS-CoV-2 3′-UTR, a negative control was run using pre-miR-125a (86 nt) in place of the full-length 3′-UTR.

To determine the efficiency of FANA-34 and FANA-760 as binding inhibitors, we performed competition experiments to the full-length 3′-UTR between the FANAs and the Cy3/DY547-tagged miRs. A sample of 150 nM Cy3/DY547-tagged miR was incubated with 250 nM of the 3′-UTR full-length construct, followed by titration of FANA-34 or FANA-760, and monitoring the fluorescence intensity at 563 nm for Cy3 or 574 nm for DY547. The normalized intensity was recorded and fitted to eq 2 for determination of the IC50 value (AAT Bioquest).^90−94^
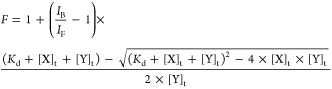
1

In eq 1, *I*_B_/*I*_F_ represents the ratio of fluorescence intensities of the bound and free RNA states (pyrC labeled TL/DIS-s2m extended or Cy3/DY547-tagged miR), and [X]_t_ and [Y]_t_ represent the concentration of the titrant miR or full length 3′-UTR, respectively. The *K*_d_ values were reported as an average of the *K*_d_ from triplicate experimentation, and the error reported is the standard deviation of those values.
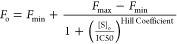
2
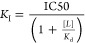
3

In eq 2, *F*_max_ and *F*_min_ represent the maximum and minimum fluorescence intensities of the experiment. *F*_o_ represents the fluorescence intensity at each data point, and [S]_o_ represents the concentration of competing ligand, in this case, the FANAs or untagged miRs. The *K*_I_ values for FANA binding were calculated using a modified Cheng–Prusoff equation (eq 3) based on the determined IC50, with [L] as the concentration of labeled miR and *K*_d_ being the experimentally derived values for the labeled miR to the full-length 3′-UTR, as determined previously.^95^ The IC50 for each FANA was reported as an average of triplicates. For *K*_I_, the reported values are the average of the calculated *K*_I_ from each IC50 value, and the error is the standard deviation of the *K*_I_ values. For evaluation of the determined IC50 and *K*_I_ values for the FANAs, the competition experiments were repeated, this time titrating unlabeled miR-760-3p, miR-34a-5p, or miR-34b-5p in place of FANA-760 or FANA-34. The IC50 values for the miRs were determined using eq 2 and compared with the IC50 values of the respective FANAs.

### UV Thermal Denaturation Spectroscopy

The complexes of miR-34a-5p/miR-34b-5p:DIS-s2m extended and miR-760-3p:3′-UTR TL were prepared by slow annealing 5 μM of each RNA, and after their cooling on the bench, MgCl_2_ (1 mM) was added, followed by incubation for an additional 1 h at 23 °C. An equal volume of mineral oil was added to the quartz cuvette to prevent evaporation. The temperature was increased from 25 to 95 °C, at a rate of 0.2 °C/min, while monitoring the absorbance at 260/275 nm. The melting curves were fit to eq 4 to the corresponding melting curves, and eq 5 was used to calculate *T*_m_.
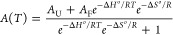
4
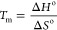
5Equation 4, which assumes a two-state model, assigns *A*_U_ as the absorbance of the unfolded complex and *A*_F_ as the folded complex.

### ^1^H NMR Spectroscopy

All 1D ^1^H NMR spectroscopy experiments of the miR-34a-5p, miR-34b-5p, and miR-760-3p binding to their respective binding site constructs were performed at 20 °C on a 500 MHz Bruker AVANCE NMR spectrometer, running the TopSpin 3.2 acquisition and processing software. RNA samples were prepared in 10 mM cacodylic acid, pH 6.5, in a 90:10 H_2_O:D_2_O ratio. The DIS-s2m extended construct at 125 μM was boiled for 5 min and snap-cooled using dry ice with ethanol. The 3′-UTR T100:TL duplex construct was prepared with both the 3′-UTR and 3′-UTR TL oligomers at 125 μM, followed by boiling for 5 min and slowly cooling for 30 min to anneal the oligomers and preform the 3′-UTR T100:TL construct. The Watergate pulse sequence was used for water suppression, and 8192 scans were collected for each spectrum.^96^ The individual miRs were also prepared at 125 μM and snap-cooled as described. For titrations, snap-cooled miR-34a-5p and miR-34b-5p were added in a 2:1 (250 μM:125 μM) ratio individually to the DIS-s2m extended, and miR-760-3p was added in the same manner to the 3′-UTR T100:TL duplex. For miR-34a-5p and miR-34b-5p the 2:1 ratio to DIS-s2m extended samples were then boiled for 5 min and slowly cooled to anneal the miRs to their respective binding sites.

## Supporting Information Available

Comparison of the isolated s2m, DIS-s2m, and DIS-s2m extended dimerization by native PAGE; Native PAGE analysis of the negative binding control of miR-132-3p to the DIS-s2m extended; ^1^H NMR spectroscopy of the miR-34a-5p and miR-34b-5p individually bound to the DIS-s2m extended; Steady-state fluorescence spectroscopy analysis of the negative binding control of miR-132-3p to the DIS-s2m extended; UV spectroscopy thermal denaturation experiments to determine the stability of the complexes formed by miR-34a-5p, miR-34b-5p, and miR-760-3p to their respective SARS-CoV-2 viral genome RNA targets; Native PAGE analysis of the miR-760-3p binding interactions to the 3′-UTR duplex mimic with the TL mutant sequence; Native PAGE of miR-34a-5p and miR-1307-3p binding to the 3′-UTR T100:TL duplex mimic; ^1^H NMR spectroscopy of the miR-760-3p binding interactions to the T100:TL duplex; Steady-state fluorescence spectroscopy of the negative control for the miR-760-3p binding interactions; MiR-132-3p as a negative control for the FANA-miRNA competition experiments (PDF)
