# Supplementary material for: Characterization of the SARS-CoV-2 Genome 3′-Untranslated Region Interactions with Host MicroRNAs
Source: ACS Omega. 2024 Aug 16;9(34):36148–64. doi: 10.1021/acsomega.4c01050 (PMC11360049; doi:10.1021/acsomega.4c01050)
Supplement: Supplementary file 1 — ao4c01050_si_001.pdf [file ao4c01050_si_001.pdf]

Characterization of the SARS-CoV-2 Genome 3'-Untranslated Region Interactions with Host microRNAs

Authors

Caleb J. Frye, Caylee L. Cunningham, Mihaela Rita Mihailescu*

***Corresponding author:** mihailescum@duq.edu

Affiliations

Department of Chemistry and Biochemistry, Duquesne University, Pittsburgh, PA, 15282, USA

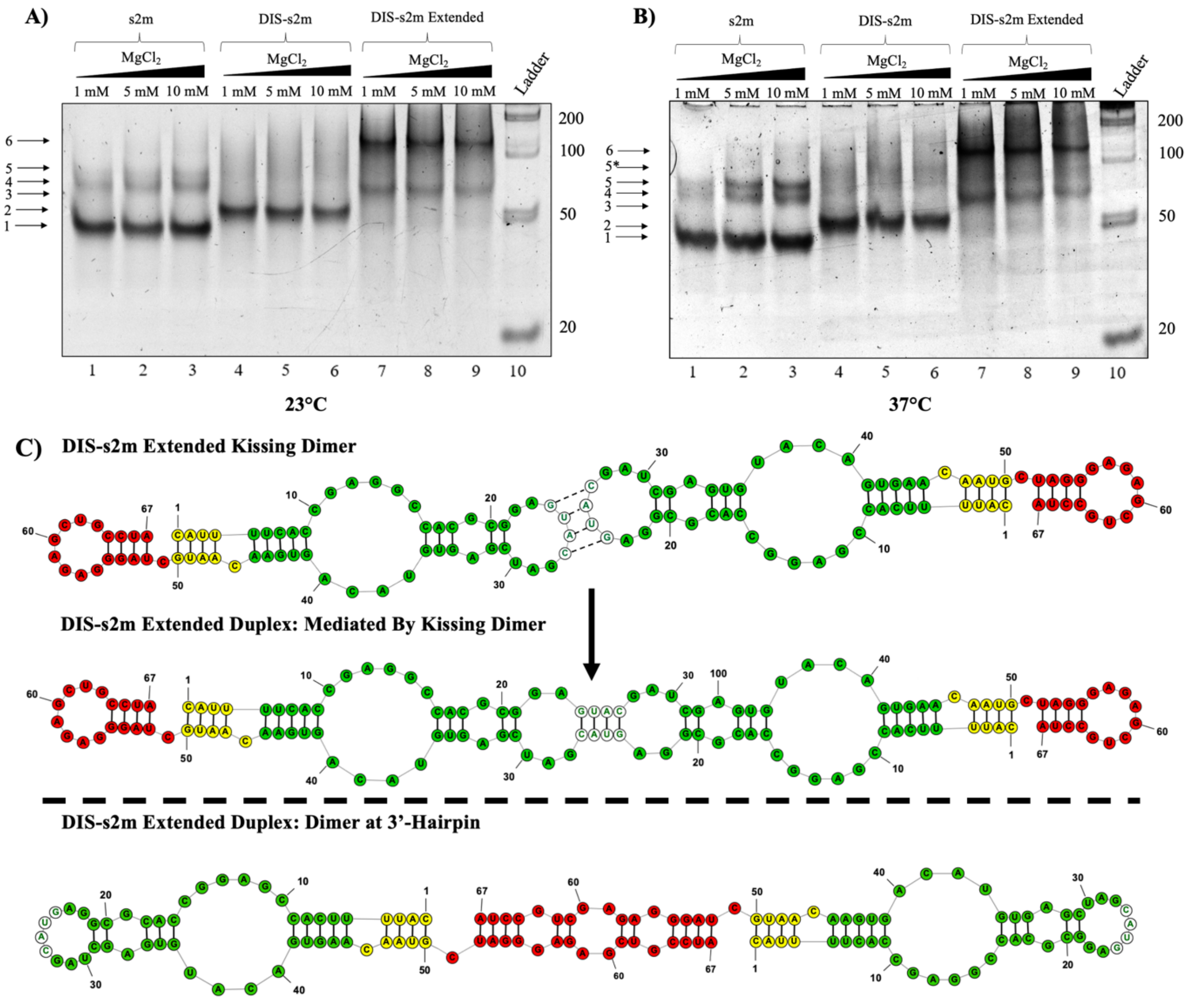

Figure S1: Comparison of the isolated s2m, DIS-s2m, and DIS-s2m extended dimerization by native PAGE. To test the dimerization of the isolated s2m, DIS-s2m, and DIS-s2m extended, samples of each oligomer were incubated with 1-10 mM $MgCl_2$ at both 23°C and 37°C in the presence of $MgCl_2$. **(A)** The isolated s2m shows the formation of both kissing dimer and extended duplex dimers (lanes 1-3, arrows 4 and 5), and extension of the motif to include its 4 lower base pairs (DIS-s2m) limits formation of these dimers (lanes 4-6). A construct containing the DIS-s2m and a 3'-tail, which contains the miR-34a/b-5p binding sites (DIS-s2m extended) forms a dimer through the dimerization of the 3'-tail (lanes 7-9, arrow 6). **(B)** Incubation at 37°C revealed greater dimer formation for all three constructs, including the DIS-s2m which shows apparent dimer formation (lanes 4-6, arrow 5*). **(C)** Structures of the DIS-s2m extended as a kissing dimer (top), a duplex mediated by the kissing dimer (middle), and a dimer mediated by the 3'-hairpin (bottom).

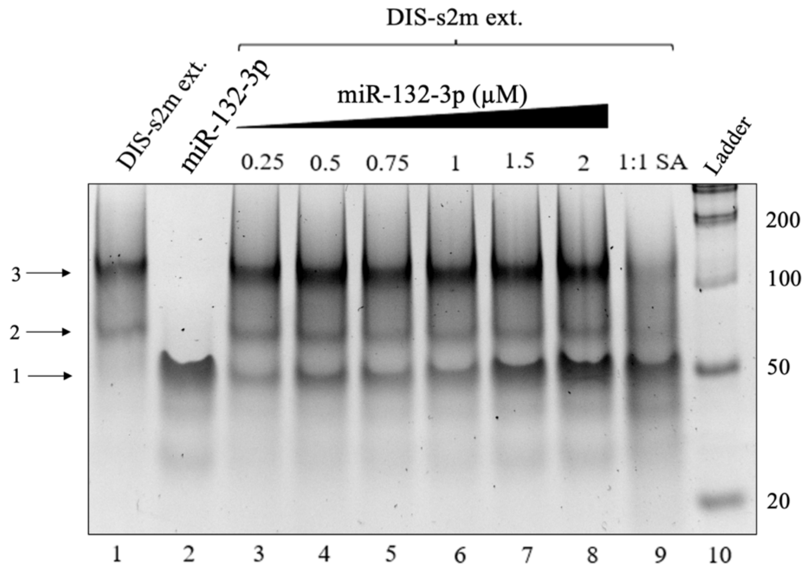

Figure S2: Native PAGE analysis of the negative binding control of miR-132-3p to the DIS-s2m extended. Titration of miR-132-3p as a specificity control to the DIS-s2m extended revealed no binding interactions, as no apparent change in the intensity of the DIS-s2m extended is observed (lanes 2-9, arrows 2 and 3) upon incremental addition of miR-132-3p.

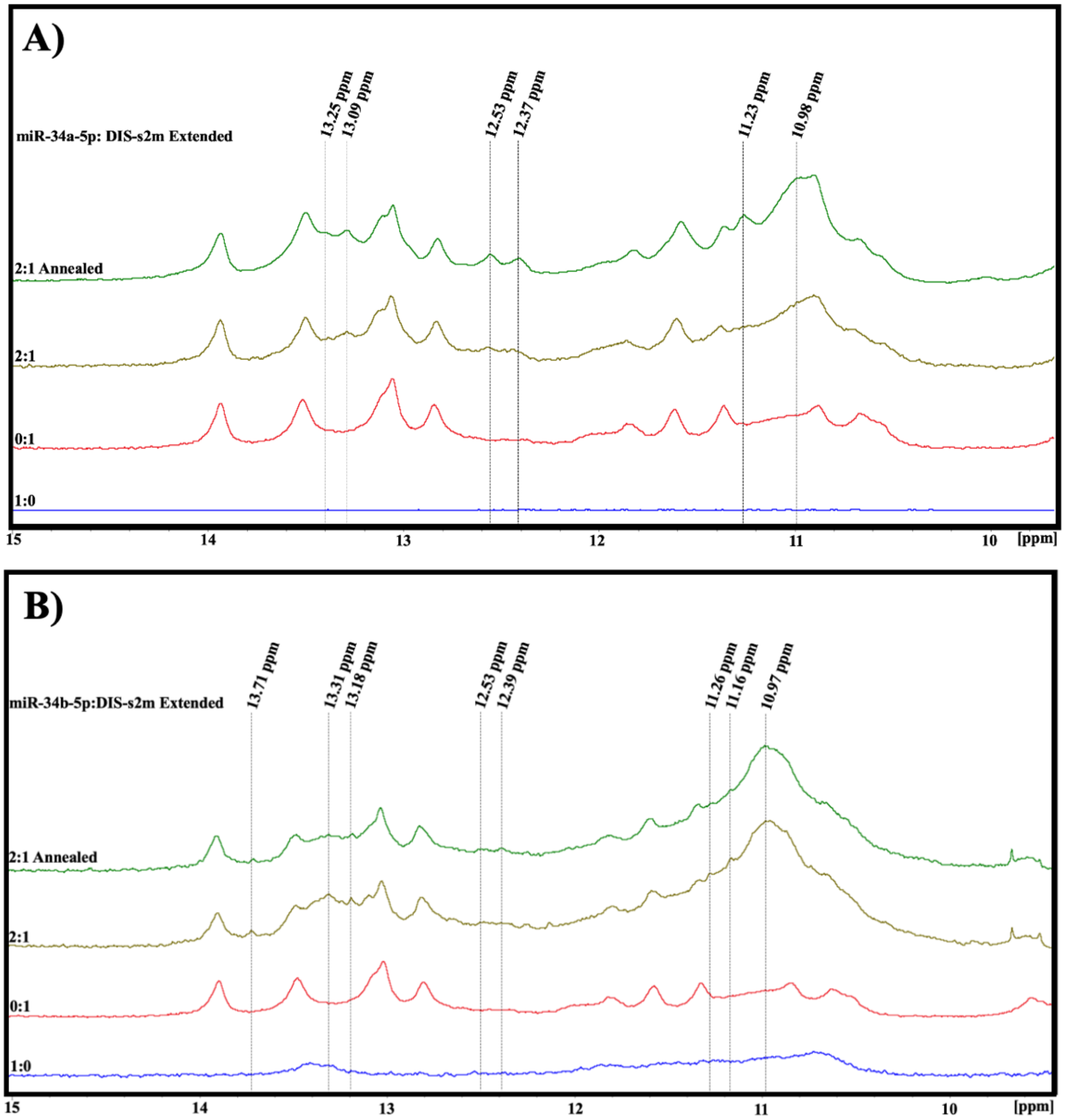

Figure S3: 1D ^{1}H NMR spectroscopy of miR-34a-5p and miR-34b-5p binding to the DIS-s2m extended. A) Titration of miR-34a-5p to the DIS-s2m extended. No imino proton resonances are present in the spectrum of the free miR34a-5p (blue) indicating that it is single stranded, whereas as expected due to its predicted extensive secondary structure, multiple resonances are present in DIS-s2m extended spectrum. Upon the incubation of miR-34a-5p with DIS-s2m extended in a 1:2 ratio DIS-s2m extended-miR new imino proton resonances appear, as indicated by the dashed lines, due to the formation of new base pairs (gold). These resonances are becoming more prominent when the 1:2 ratio DIS-s2m extended-miR-34a-5p sample was slow annealed to promote forced binding (green). Additionally, comparison of the spectrum for the miR-bound complex (gold and green) compared to that of the DIS-s2m extended itself (red) reveals that the majority of the secondary structure of the DIS-s2m extended is retained, as no significant loss of original resonances is evident. **B)** Similar experiments were performed with miR-34b-5p and DIS-s2m extended and new imino proton resonances highlighted by the dashed lines were observed, with no significant loss of original resonances. For both **A)** and **B)**, samples in which snap-cooled miR is added in 1:2 stoichiometric ratio and incubated on the bench are shown in mustard, followed by slow annealed samples at a 1:2 ratio in green.

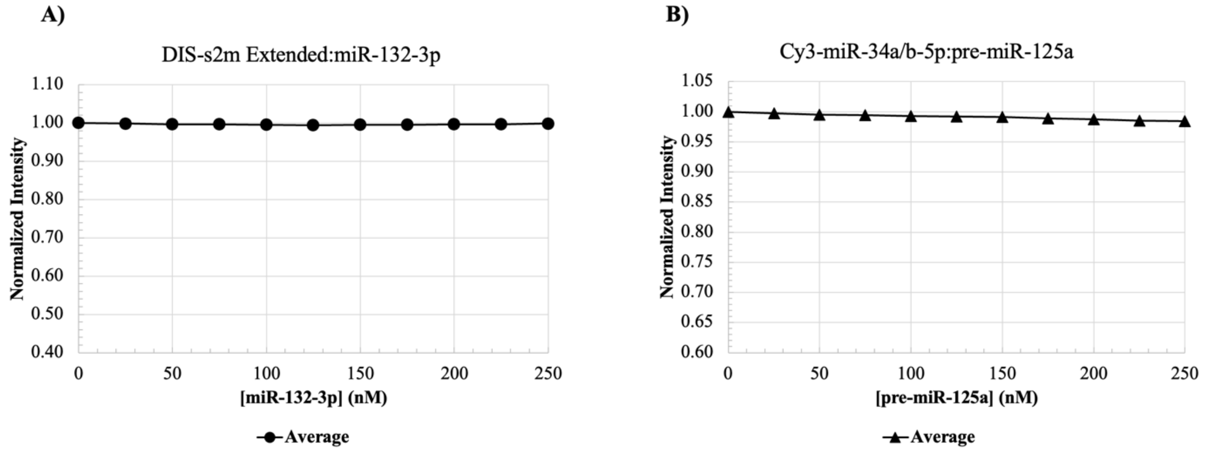

Figure S4: Steady-state fluorescence spectroscopy analysis of the negative binding control of miR-132-3p to the DIS-s2m Extended. (A) The titration of miR-132-3p incrementally to the DIS-s2m extended revealed no change in fluorescent intensity. **(B)** Titration of pre-miR-125a to the Cy3-tagged miR-34a/b-5p oligomers revealed no change in fluorescent intensity, consistent with prior controls in demonstrating sequence specificity of these interactions for the target miRNAs.

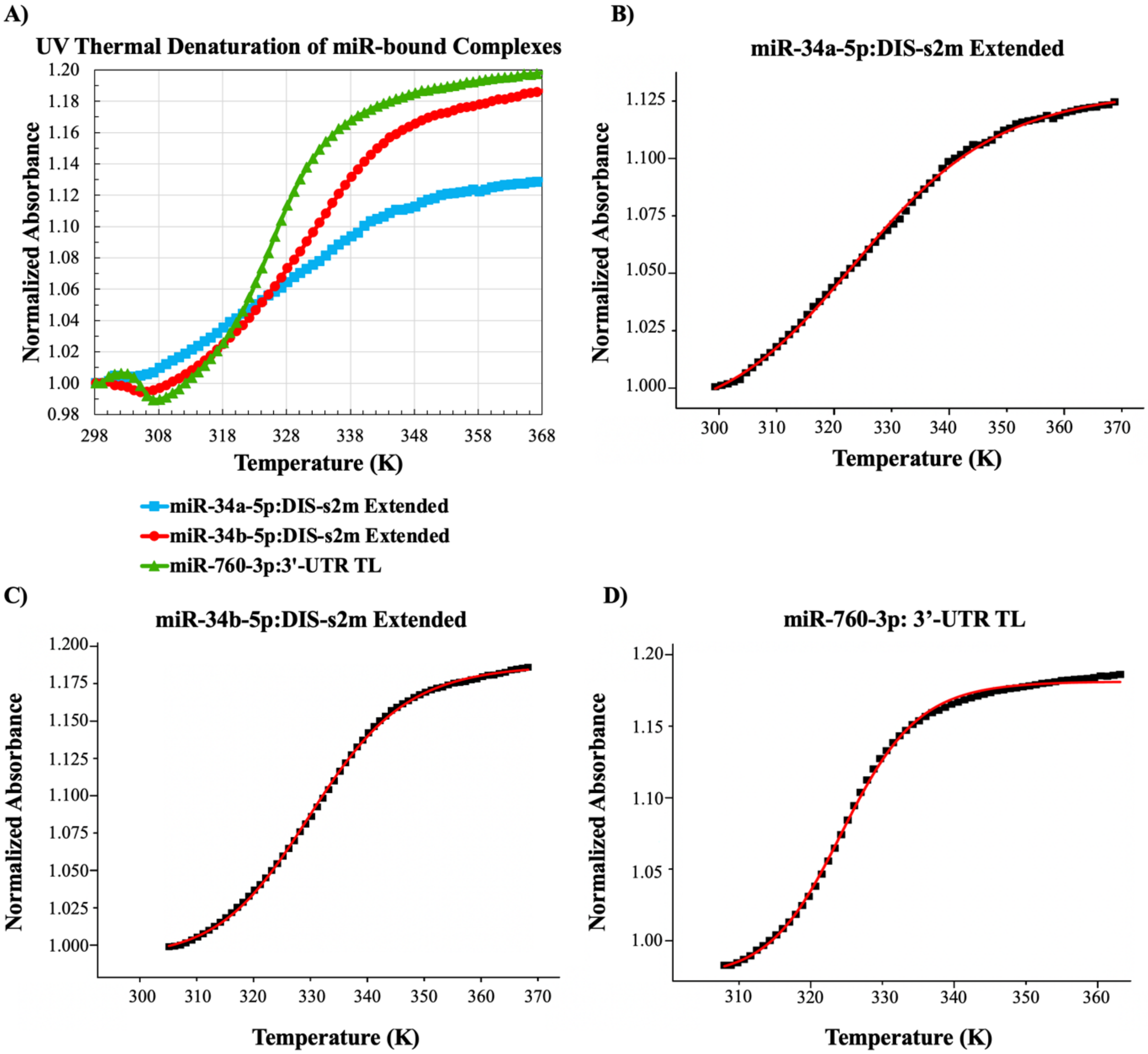

Figure S5: UV spectroscopy thermal denaturation experiments to determine the stability of the complexes formed by miR-34a-5p, miR-34b-5p, and miR-760-3p to their respective SARS-CoV-2 viral genome RNA targets. For the miR-34a-5p and miR-34b-5p complexes, the absorbance changes were monitored at 260 nm, whereas for the miR-760-3p the absorbance change was monitored at 275 nm, as the temperature was increased from 25°C to 95°C. The hyperchromic transitions were fit to equation 4 to determine the T_m for each complex. **(A)** Overlay of the three thermal denaturation curves. Fit curves for **(B)** the miR-34a-5p:DIS-s2m extended complex, **(C)** the miR-34-5p:DIS-s2m extended complex, and **(D)** the miR-760-3p:3'-UTR TL complex.

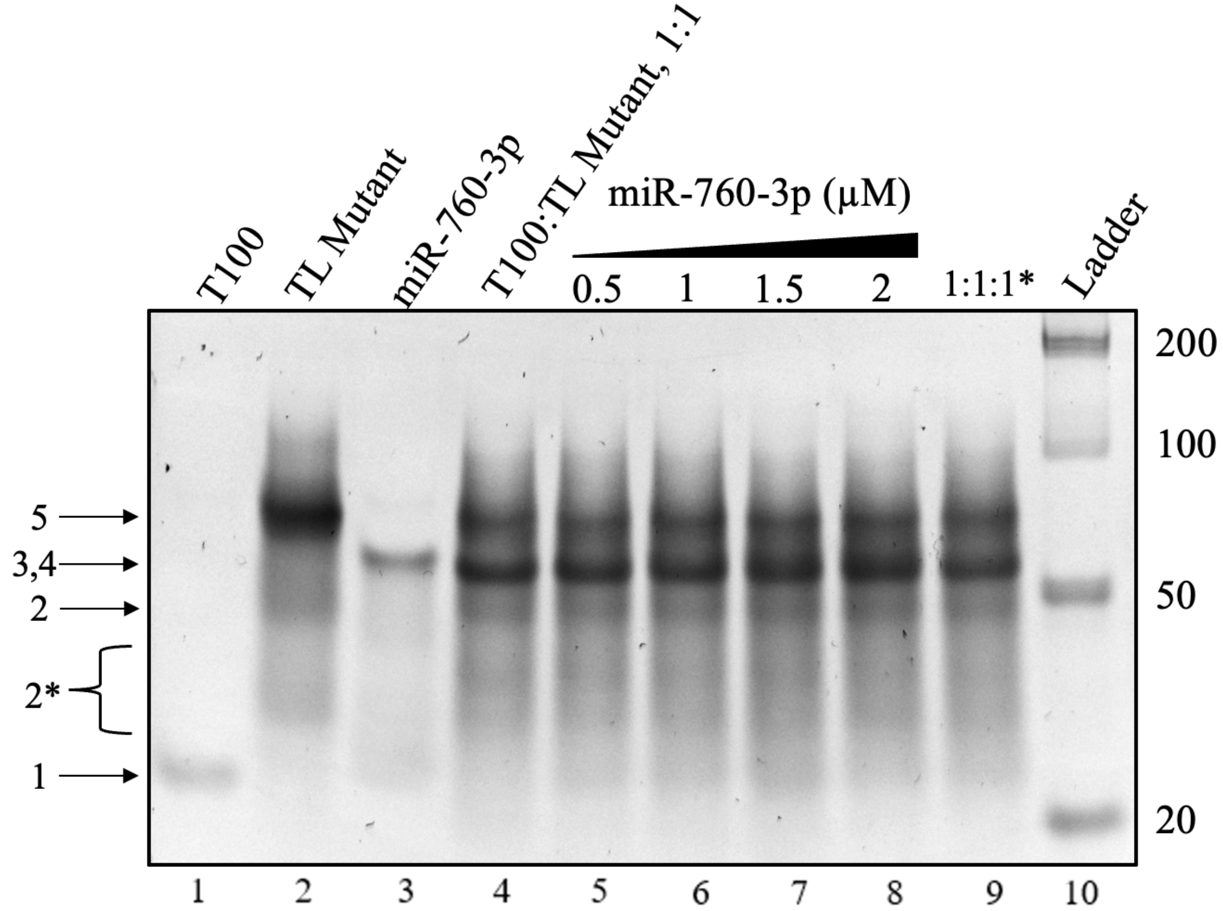

Figure S6: Native PAGE analysis of the miR-760-3p binding interactions to the 3'-UTR duplex mimic with the TL mutant sequence. To confirm the specific interactions of miR-760-3p to the exposed bulge formed in the 3'-UTR T100:TL duplex mimic, the miR-760-3p binding experiments were repeated with a mutated bulge on the TL. The T100 monomer (lane 1, arrow 1) and TL mutant sequence (lane 2, arrows 2 and 5) were slow annealed forming the 3'-UTR T100:TL duplex mimic (lane 4, arrow 4). Upon titration of miR-760-3p, no binding to the 3'-UTR T100:TL duplex mimic was observed. Several smaller molecular weight bands were observed (lanes 2-9, bracket 2*), attributed to degradation products of the TL mutant and miR-760-3p sequences.

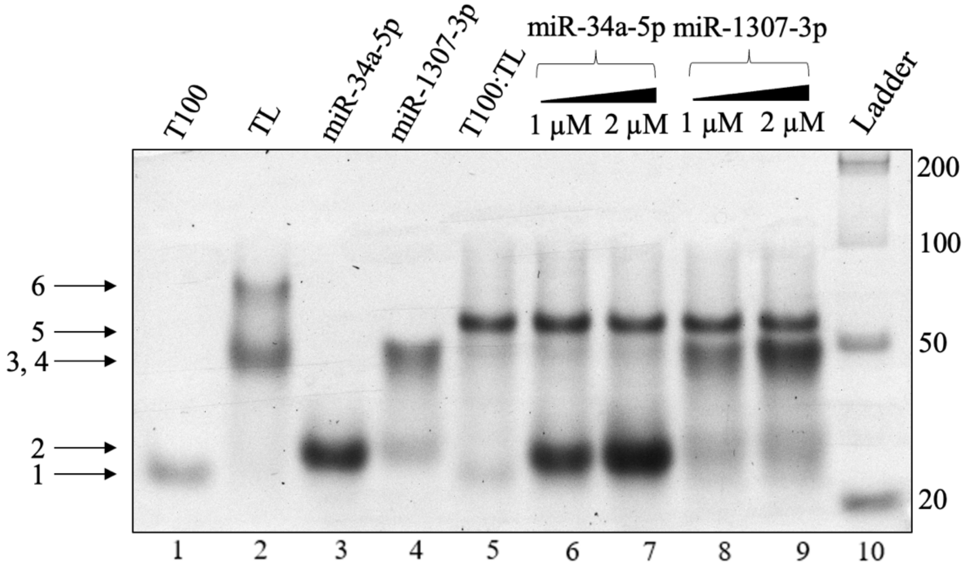

Figure S7: Native PAGE of miR-34a-5p and miR-1307-3p binding to the 3’-UTR T100:TL duplex mimic. To establish sequence specificity of the 3’-UTR T100:TL duplex mimic for miR-760-3p, miR-34a-5p (lanes 3, 6, and 7, arrow 2) and miR-1307-3p (lanes 4, 8, and 9, arrow 4) were used as controls. Lane 1: free T100 (arrow 1); lane 2: TL (arrows 3 and 6). Preformation of the 3’-UTR T100:TL duplex mimic (lanes 4-9, arrow 5), followed by titration of either miR-34a-5p or miR-1307-3p, did not result in the appearance of any higher molecular weight complex bands. No change in intensity of the 3’-UTR T100:TL duplex band (lanes 4-9, arrow 5) was observed.

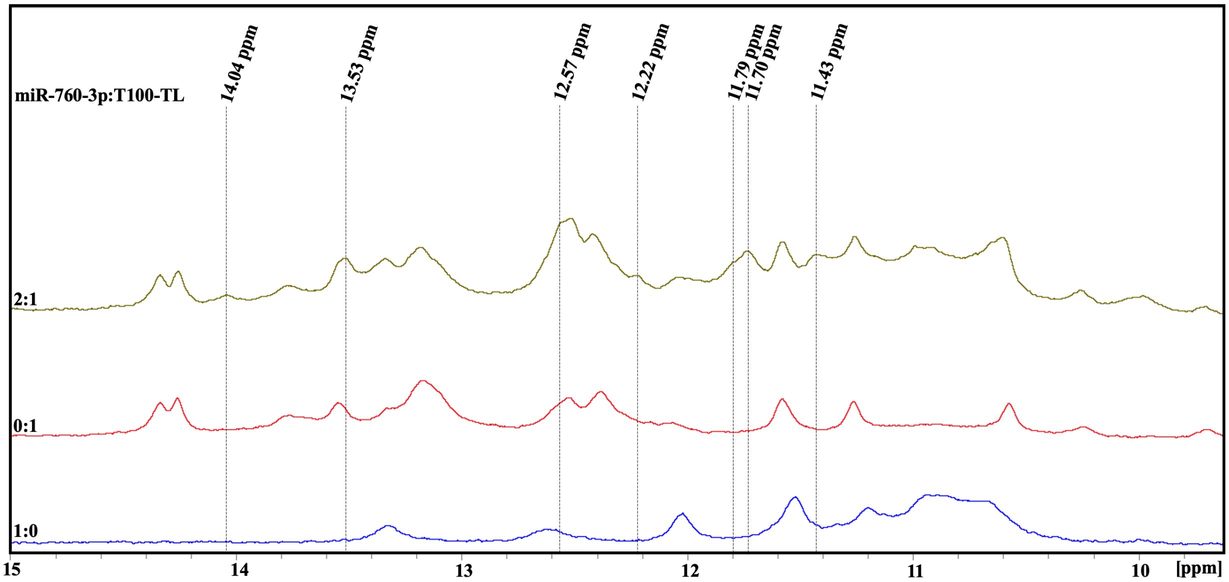

Figure S8: 1D ^{1}H NMR spectroscopy of the miR-760-3p binding to the 3'-UTR T100:TL duplex. Further analysis of the miR-760-3p binding interactions with the 3'-UTR T100:TL duplex was performed by analyzing the imino proton resonances obtained through 1D ^{1}H NMR spectroscopy. Upon titration of miR-760-3p in a ratio of 1:2 for T100-TL:miR-760-3p (mustard), we observe the formation of new imino proton resonance indicative of new base pair formation. We also observe no loss of resonances upon addition of the miR-760-3p to the 3'-UTR T100:TL duplex, suggesting conservation of the original structure and supporting that miR-760-3p binding does not displace T100 from the preformed 3'-UTR T100:TL duplex.

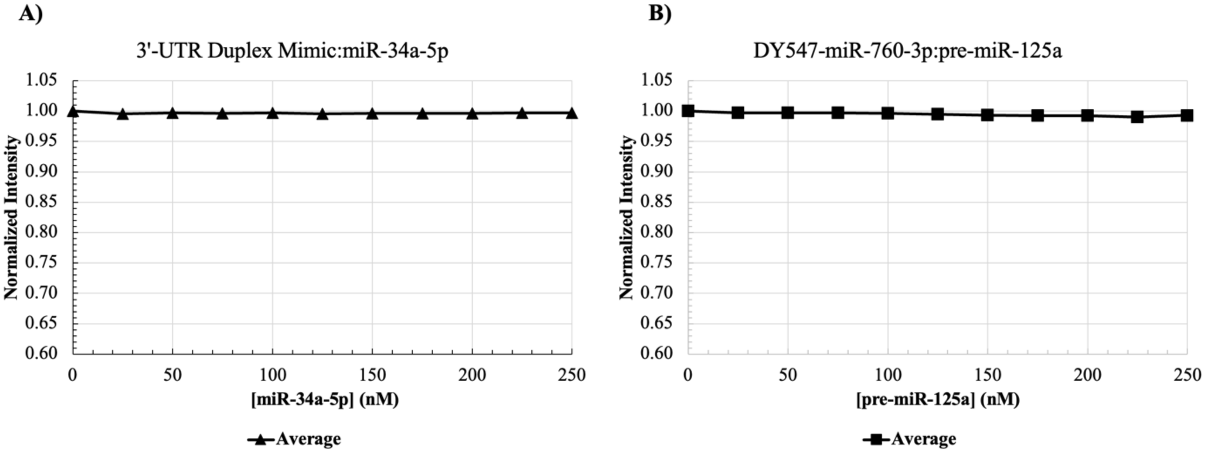

Figure S9: Steady-state fluorescence spectroscopy of the negative control for the miR-760-3p binding interactions. (A) Titration of miR-34a-5p was performed as a negative binding control for the miR-760-3p binding experiments. Upon incremental addition of miR-34a-5p, no significant change was observed in the fluorescence intensity of the pyrC-tagged 3'-UTR T100:TL duplex mimic. **(B)** For the full-length 3'-UTR experiments, pre-miR-125a was titrated to DY547-miR-760-3p as a negative control. No significant decrease in fluorescence intensity was observed, indicating specificity of the 3'-UTR for miR-760-3p.

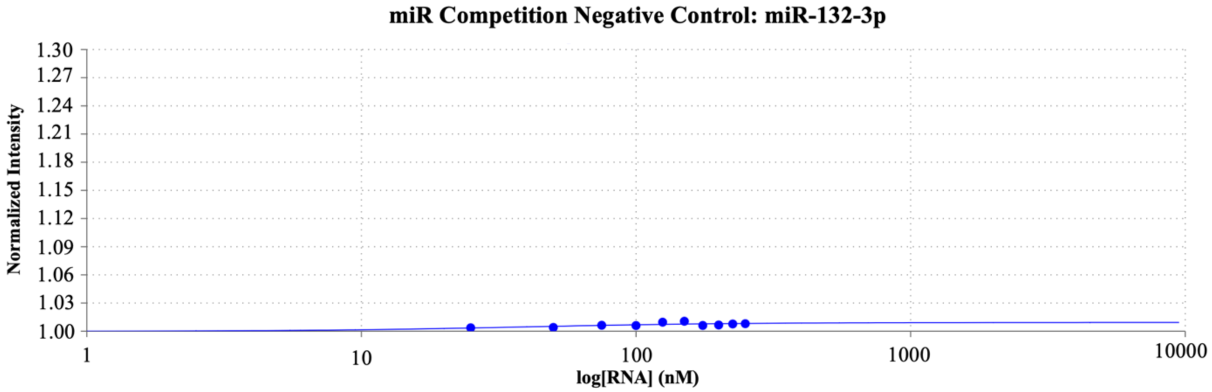

Figure S10: MiR-132-3p as a negative control for the FANA-miRNA competition experiments. To demonstrate the specific activity of FANA-760 and FANA-34 in their competition with the wild-type microRNAs, miR-132-3p was used as a negative control. The fluorescent intensities were normalized to the initial intensity, in which there is 150 nM of miRNA to 250 nM of 3'-UTR. Where previously the FANAs demonstrate a gain in fluorescence intensity upon titration, no significant regain fluorescence intensity was observed upon the addition of miR-132-3p.
